# Confinement‐Induced Metastable Supramolecular Assembly: Dynamic Built‐in Electric Field Driving Deep Mineralization of Organic Pollutants

**DOI:** 10.1002/advs.202506797

**Published:** 2025-06-05

**Authors:** Xiaoqing Cao, Kaixiang Liang, Feifei Peng, Xianggui Kong, Wenying Shi

**Affiliations:** ^1^ State Key Laboratory of Chemical Resource Engineering Beijing University of Chemical Technology 15 Beisanhuan East Road Beijing 100029 P. R. China

**Keywords:** confinement, internal electric field, metastable supramolecular assembly, photogenerated carrier separation

## Abstract

Constructing built‐in electric fields (IEF) in homomeric supramolecular assembly (SA) offers an exciting research avenue. To boost dipole, the introduction of a functional side chain in the monomer is essential. Unfortunately, this disrupts the monomer planarity, inevitably increasing intermolecular distance, which is contradictory to achieving high IEF. Here, by means of the confinement of layered double hydroxide (LDH), various monomers (naphthalene and perylene diimide derivatives) successfully form metastable SA with smaller intermolecular distances than in crystals. The IEF enhancement value can reach 11.8 times, enabling a 71.52% charge separation efficiency and a 78.10% total organic carbon (TOC) removal rate in phenol degradation, presenting state‐of‐the‐art results. Mechanism elucidates LDH confinement overcomes the energy barrier associated with the ordered and close packing of monomers, moving monomer away from the thermodynamic equilibrium state. The universality of this approach will pave the way for exploring other multifunctional monomers, thereby fostering advancement in material science, chemical synthesis, and photo‐conversion.

## Graphical abstract

Utilizing the 2D confined space of layered double hydroxide (LDH), living supramolecular assembly (LSA) are successfully prepared with smaller intermolecular distances than in crystals. The IEF enhancement value can reach 11.8 times, which induces a charge separation efficiency of 71.52%.

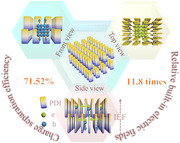

## 1 Introduction

The emergence of the built‐in electric fields (IEF) provides a novel research avenue in performance improvement of photoelectric devices due to the promoting charge‐carrier separation.^[^ ^1^, ^2^, ^3^, ^4^, ^5^, ^6^ ^]^ Generally, the nonuniform charge distribution in the single‐component material or at the heterointerface of the hetero‐component material induce IEF, which first appeared in inorganic materials.^[^ ^7^, ^8^, ^9^, ^10^, ^11^, ^12^, ^13^, ^14^ ^]^ To achieve IEF, inorganic materials must have a non‐centrosymmetric crystal structure, meaning that its crystal lattice lacks inversion symmetry.^[^ ^15^ ^]^ To satisfy this condition, scientists often need to carefully engineer the crystal structure, which greatly limits the universality and practical application. By contrast, organic materials offer a wide range of chemical structures and functional groups,^[^ ^16^, ^17^ ^]^ allowing for precise tuning of their energy levels, bandgaps, and charge transport properties, subsequently facilitating the development of IEF.^[^ ^18^, ^19^, ^20^ ^]^ Thus, in 2000, Nicoletta's group first proposed the IEF in an organic material polymethylmethacrylate by applying an external voltage.^[^ ^21^ ^]^ Unfortunately, the use of external voltage inevitably brings some additional energy consumption, limiting the driving effect of IEF on the carrier transport. Therefore, in 2014, Zhu's group assembled Poly‐3‐hexylthiophene particles on carbon nitride (g‐C_3_N_4_) nanoplates, exploiting the inherent properties of hetero‐component to break through the bottleneck of relying on external energy sources and realize the high IEF.^[^ ^22^ ^]^ This provoked a shift toward the IEF construction of organic materials. However, the challenge for the successful acquisition of heterogeneous structures lies in the strict matching of the energy bands of the two components, which greatly limits the universality of this method. In order to avoid the above problems, it is a good choice to construct the IEF in the supramolecular assembly (SA) composed of the same monomers.^[^ ^23^, ^24^, ^25^, ^26^ ^]^ Thus, in 2018, Wang's group achieved high IEF in porphyrin supramolecular nanomaterials by the non‐covalent interaction.^[^ ^27^ ^]^ Generally, to obtain a high IEF, the introduction of side chain functional groups on monomers to increase the molecular dipole is used. Unfortunately, the introduction of functional groups disrupts the planarity of the molecule and inevitably leads to an increase in the distance between adjacent monomers, subsequently the decrease in the IEF. This is because the intermolecular distance is inversely proportional to the macroscopic dipole moment of the molecule in SA systems.^[^ ^28^ ^]^ Therefore, the key to solving this problem lies in finding suitable assembly methodologies and pathways to force the intermolecular distance to be reduced below the conventional distance, so as to enhance the IEF.

The spatial confinement has the ability to regulate intermolecular distances and orientation by enhancing intermolecular forces.^[^ ^29^ ^]^ This process moves monomers away from the isotropic stacking arrangement of the thermodynamically stable state, and leading to different results than in the non‐confined domain condition. For example, in the organelle confined environment, a self‐assembled tighter structure of chlorophyll and protein promote their light‐trapping ability.^[^ ^30^ ^]^ The confined space of a protein cage or self‐assembled capsule make the interaction between reactants and catalysts stronger, which effectively facilitate organocatalytic reactions.^[^ ^31^, ^32^, ^33^, ^34^, ^35^ ^]^ In the capillary confined environment, a self‐assembling ordered structure composed of amino acids accelerated the adhesion and proliferation of cell.^[^ ^36^ ^]^ The aforementioned phenomena have provided important clues that the spatial confinement environment has the ability to reduce the intermolecular distances, achieving the tightly molecular stacking.

**Figure 1:**
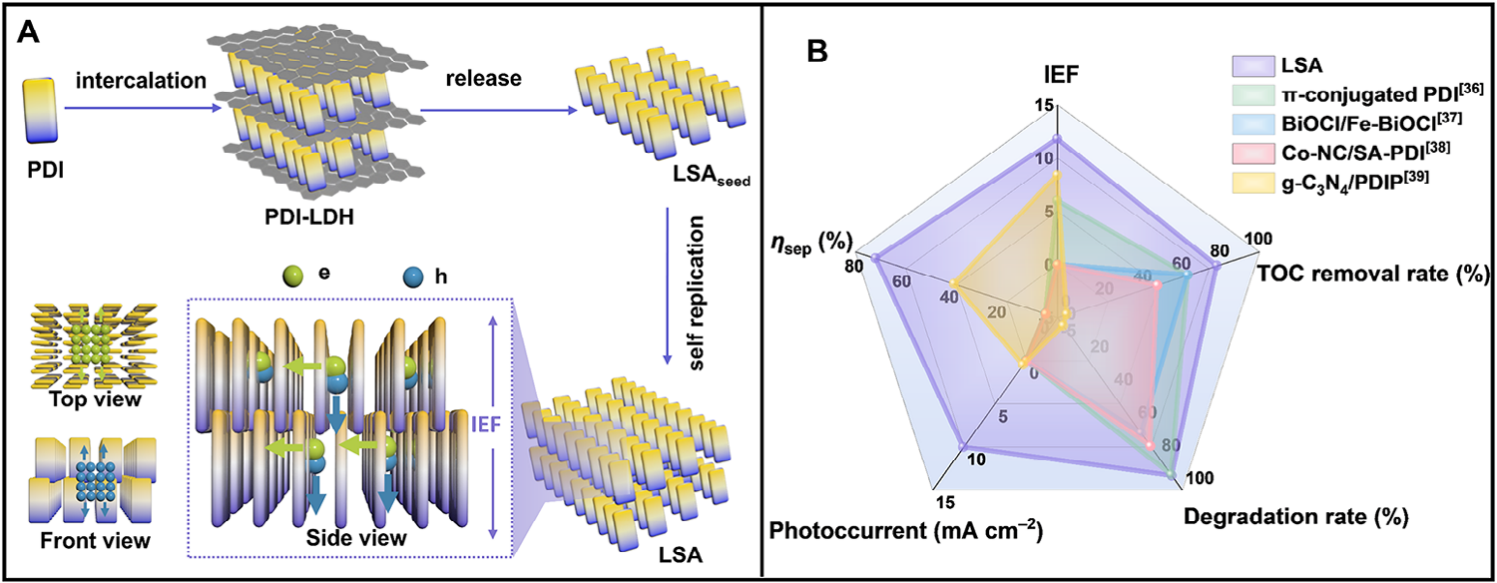
A) Schematic presentation for the formation process of LSA and illustration of the separation and migration of electrons and holes in LSA. B) Comparison of IEF intensity, *η*_sep_, photocurrent, degradation rate and TOC removal rate of LSA and other photocatalysts.^[^ ^38^, ^39^, ^40^, ^41^ ^]^

Here, we choose the organic molecule N,N'‐di(propanoic acid)‐perylene‐3,4,9,10‐tetracarboxylic diamide (PDI) as the model monomer (**Figure** **1**A). Theoretically, the SA composed of the non‐completely flat monomers must have an intermolecular distance greater than 0.34 nm owing to hydrogen bonding as the driving force for assembly, implying the IEF of SA must be less than that of crystal.^[^ ^37^ ^]^ Unexpectedly, after experiencing the 2D confinement of layered double hydroxide (LDH), PDI assembly (named living SA, LSA_seed_, see “Methods” for details) showed an enhanced IEF by ≈11.8 times relative to its crystal, which induces a charge separation efficiency (*η*_sep_) of 71.52%. Exhibiting a phenol degradation rate constant of 1.28 h^−1^ (21.3 times faster than PDI crystal) alongside 78.1% total organic carbon (TOC) removal efficiency (7.2‐fold enhancement over the PDI crystal), presenting a state‐of‐the‐art result (Figure 1B).^[^ ^38^, ^39^, ^40^, ^41^ ^]^ Due to the confinement effect of LDH, the LSA_seed_ has the ability of living growth, and the resulting longer and ordered π‐π stacking is beneficial to enhancing the dipole and IEF, thus promoting the separation of photogenerated hole‐electron pair. The electrons tend to remain in the core of LSA and transport in the direction of the *π*‐*π* stacking; the holes migrate outward along the *π*‐*π* plane of the PDI molecule. This work underlines the great potential of SA formed in LDH confinement and provides new perspectives for the design and development of efficient organic photocatalysts.

## 2 Results and Discussion

### 2.1 The Compact *π*‐*π* Stacking Induced by Confinement

**Figure 2:**
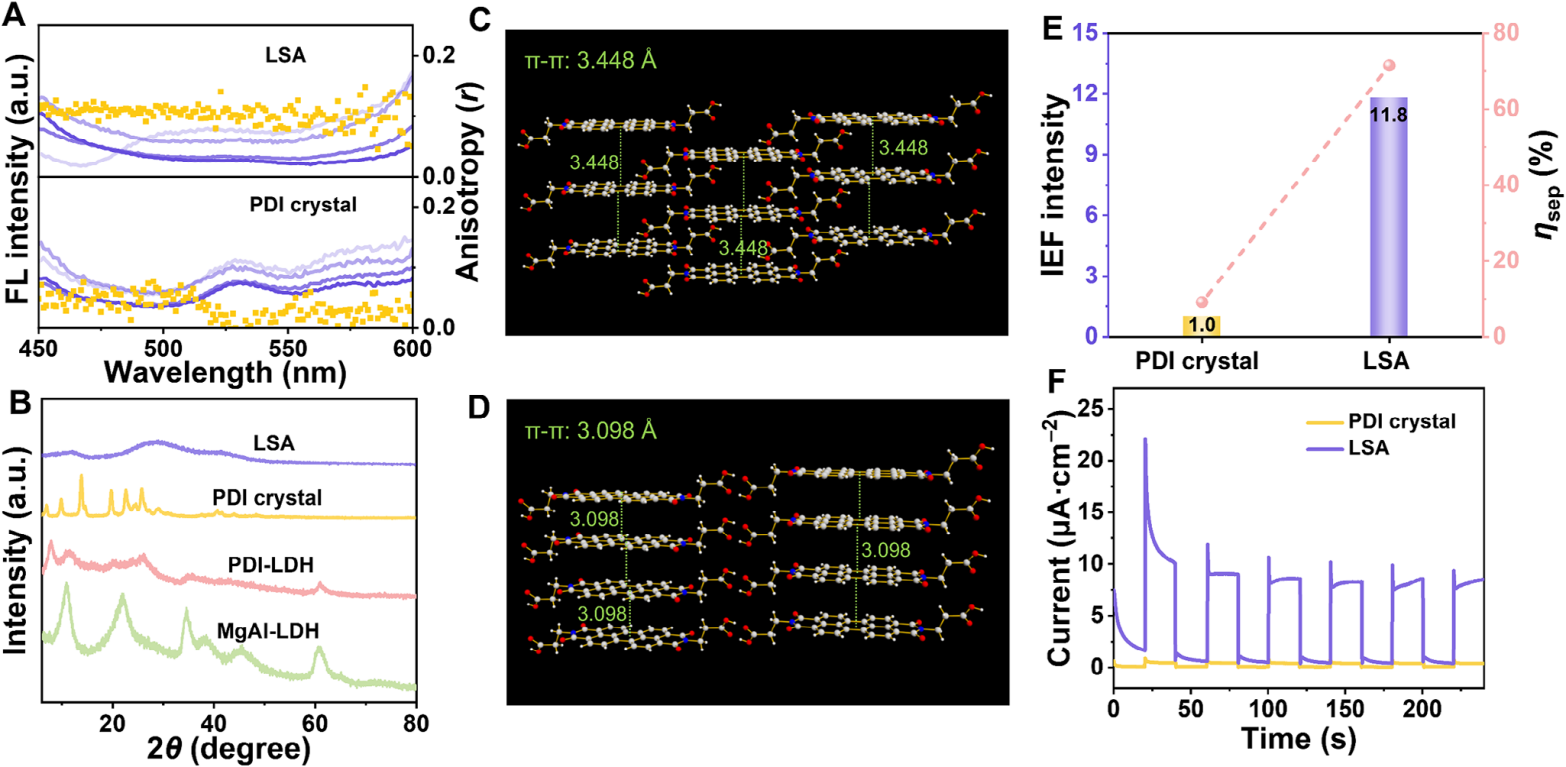
Structure and optoelectronic properties of PDI crystal and LSA. A) Anisotropic profiles of LSA and PDI crystal. B) XRD patterns of MgAl‐LDH, PDI‐LDH, LSA and PDI crystal. The *π‐π* stacking diagram of C) PDI crystal and D) LSA. E) The relative IEF intensity and charge separation efficiency of LSA and PDI crystal. F) Photocurrent of the LSA and PDI crystal.

Confinement‐mediated assembly of PDI molecules including intercalation and release. After experiencing the confinement space of LDH, the stacking mode of PDI undergoes significant changes compared with PDI crystal. After intercalating, the type of PDI stacking in LSA is J‐type *π‐π* stacking, which was confirmed by wavelength red shift in fluorescence (FL) emission to 644 nm compared with PDI molecules of 580 nm (Figure S1, Supporting Information).^[^ ^42^, ^43^ ^]^ LSA presents a 2D topological arrangement with high anisotropy imprinted from the LDH structure. As testified by FL polarization spectra (**Figure** **2**A), LSA shows a higher anisotropic value (*r* = 0.110). In contrast, in the crystal structure, the PDI molecules are arranged in a 3D periodic topology and appear isotropic with a lower *r* value (0.024) on a macroscopic level. Stronger hydrogen bonding interactions are present in LSA as evidenced by the shift of the C ═ O absorption peak of LSA to lower wavenumber in the FTIR spectra (Figure S2, Supporting Information).

The precise changes of PDI in stacking type can be monitored using X‐ray diffraction (XRD, Figure 2B). The successful insertion of PDI monomer into the confinement space of the LDH can be confirmed by an increased *d*_003_ to 11.4 Å compared to MgAl‐LDH. The size of LDH is not significantly affected by the intercalation process of PDI (Figure S3, Supporting Information). PDI presents a monolayer oblique arrangement, as evidenced by the smaller *d*‐spacing (1.14 nm) of PDI‐LDH compared to the length of PDI molecule (2.18 nm, Figure S4, Supporting Information). The arrangement difference of PDI molecules in LSA and the crystal can also be evidenced from XRD patterns, where LSA only show broad diffraction peaks, while PDI crystal shows strong and narrow diffraction peak. The distinctly different structural feature amply demonstrates LSA is an amorphous structure, rather than crystal. In addition, this can also be demonstrated from the perspective of formation conditions. The LSA could be prepared with low concentrations. Specifically, the concentration here is obtained by converting PDI‐LDH, which is far below supersaturation concentration of PDI ([PDI] ≫ 40 mm, Figure S5, Supporting Information). This result ruled out the possibility that LSA were formed through a typical crystallization process. In addition, LSA shows a reversible formation feature, which can be proved by disassembly via increase of good solvent and re‐assembly via increase of poor solvent, which can be monitored by FL spectra (Figures S6 and S7, Supporting Information). By increasing the ratio of propionic acid, LSA disassembled to LSA_seed_, as confirmed by FL wavelength shift from 660 to 647 nm; by increasing the ratio of CH_3_OH to propionic acid/CH_3_OH (1:3 v/v), LSA_seed_ quickly re‐assemble to LSA, as confirmed by FL wavelength shift from 647 to 661 nm. In theory, the intermolecular distance in a SA system should exceed the crystal lattice distance because of the non‐covalent interactions that establish the SA structure. Unexpectedly, the confinement‐mediated assembly results in tighter stacking, which makes π‐π distance in LSA even smaller than that in a crystal. As confirmed by XRD, the π‐π stacking diffraction peak of LSA shift to a higher angle at 2*θ* = 28.8° compared with crystal of 25.8° (Figure 2C,D). Subseqently, the distances between adjacent molecules of LSA and crystal is *d* = 3.098 and 3.448 Å, respectively.^[^ ^44^ ^]^

### 2.2 Enhanced Internal Electric Field (IEF) by Compact *π‐π* Stacking

The distance between adjacent molecules and the IEF of SA is inversely related, where closer proximity usually results in a stronger IEF. Because when adjacent molecules are closely arranged, their electron clouds may overlap, leading to enhanced intermolecular interactions such as dipole‐dipole interactions and subsequently enhanced IEF. To quantitatively reflect the intensity of IEF, the photo‐generated charge density and open circuit potential (OCP) were monitored according to Equation (1).

(1) $$F_{S}={\left(\frac{-2V_{s}\rho }{\varepsilon \varepsilon_{0}}\right)}^{1/2}\;$$

where *F*_s_ is the magnitude of the internal electric field, *V*_s_ is the surface photovoltage, *ρ* is the surface charge density, Ɛ for low frequency dielectric constant, Ɛ_0_ for the permittivity of free space. Since the Ɛ and Ɛ_0_ are two constants, the *F*_s_ mainly decided by the surface voltage and charge density. Compared with PDI crystal, the photogenerated *ρ* increases from 0.67 to 39.44 µC cm^−2^, and the OCP increases from 126 to 301 mV (Figures S8 and S9, Supporting Information). Thus, the relative IEF of LSA is 11.8 compared with PDI crystal (Figure 2E), which facilitates charge separation and high photoelectric signals. The charge separation efficiency of the LSA can reach *η*_sep_ = 71.52%, which is 12.8 times that of PDI crystal with *η*_sep_ = 9.12% (Figure S10, Supporting Information). Accordingly, the photocurrent value of LSA (10.07 µA) is increased by 22 times compared with PDI crystal with 0.45 µA (Figure 2F).

### 2.3 Enhanced IEF by the Length of *π‐π* Stacking

**Figure 3:**
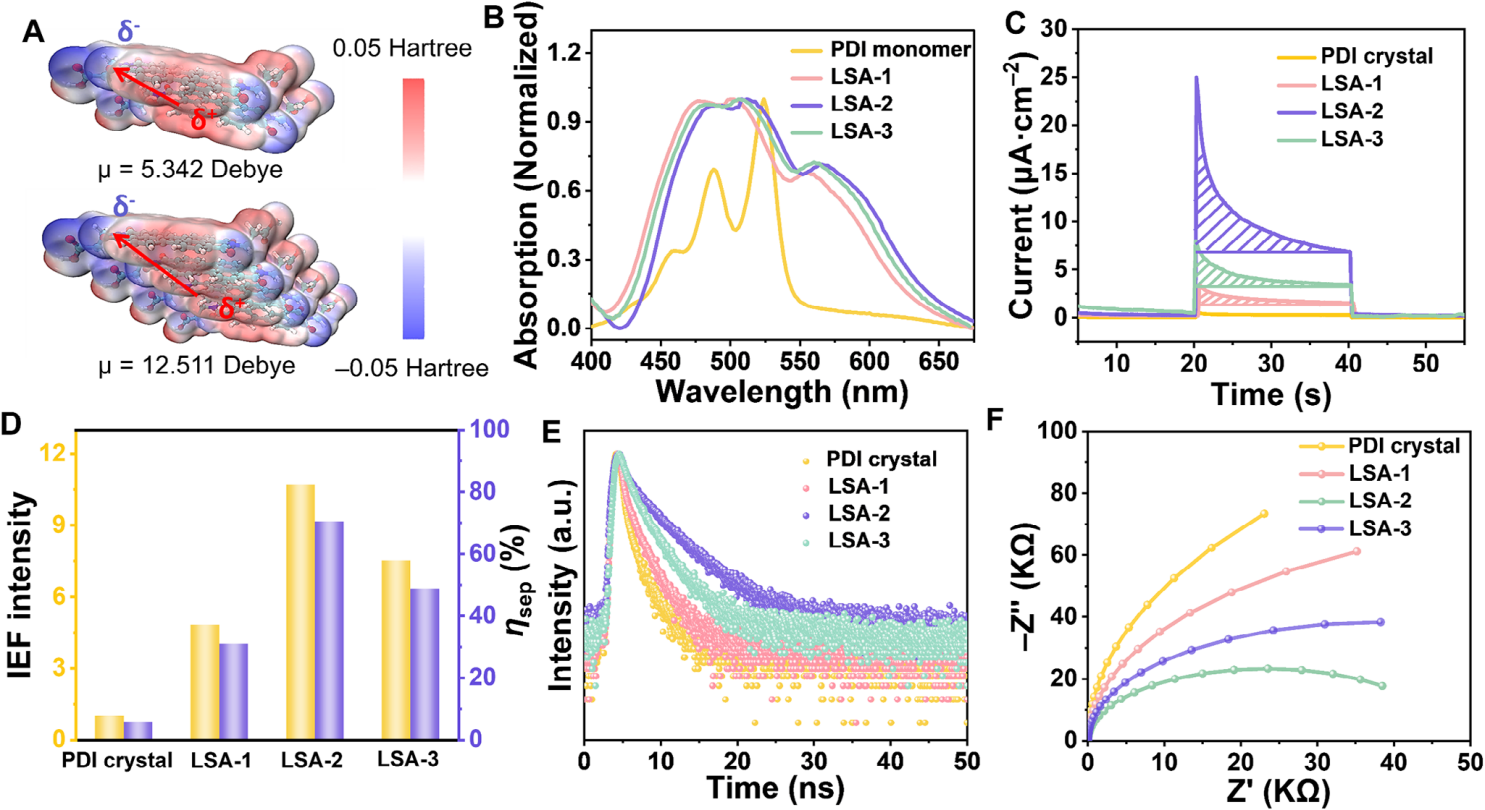
LSA with size‐dependent IEF and photovoltaic properties. A) Surface electrostatic potential of PDI dimer and PDI tetramer. B) UV–vis spectra of PDI monomer, LSA‐1, LSA‐2 and LSA‐3. Photo‐induced charge transport properties of PDI crystal, LSA‐1, LSA‐2, and LSA‐3: C) the photocurrent density, D) the relative IEF intensity and charge separation efficiency, E) time‐resolved fluorescence spectra, and F) electrochemical impedance spectra.

Based on the distribution and interaction of the electric field, as the size of the LSA increases, the distribution of the IEF throughout the system becomes more uniform and stronger. Density functional theory (DFT) calculations demonstrate a substantial elevation in the molecular dipole, escalating from 0, 5.342, and 9.962 to 12.511 Debye with the monomer number increasing from one, dimer, trimer, and tetramer (Figure S11, Supporting Information). The details of model construction are listed in the methods section, which are referred to XRD, ICP‐MS and elemental analysis results (Tables S1 and S2, Supporting Information). It can be seen the surface electrostatic potential difference of the molecule is mainly caused by the oxygen atom at the acid anhydride and the carbon in the conjugated ring (**Figure** **3**A). Therefore, the negative charge is mainly concentrated at the position of the terminal oxygen, and the positive polarity is exhibited in the ring.^[^ ^45^ ^]^ When the assembly changes from dimer to tetramer, due to the increased intermolecular force and the asymmetric structure of molecules, the positive and negative center deflection is obviously shifted. Due to the limitation of the calculation program, we only calculated up to the tetramer. With the increase of the assembled number of PDI molecules, the polarization degree of electrostatic potential increased obviously. In reality, the number of assemblies is much larger than this value, indicating that the LSA has a high degree of polarization, which is conducive to obtaining a great IEF.

In order to further analyze the spatial distribution of electron‐hole of LSA and monomer upon excitation, electron‐hole excitation analyses were carried out by using UV–vis absorption spectroscopy (Figure 3B). The PDI monomer exhibits prominent peaks at 524, 488, and 459 nm, corresponding to the electronic transitions of 0‐0, 0–1, 0–2, respectively.^[^ ^46^, ^47^ ^]^ In contrast, the LSA shows a noticeable red shift and broadening in absorption bands reminiscent of a charge transfer (CT) transition, characterized by large indexes of spatial distribution for electrons and holes.^[^ ^23^ ^]^ The excited states favorable for CT have also changed, from the S2 state in monomer to the S1 state in LSA. Furthermore, the polarization effect induced by long‐range *π‐π* stacking and the well‐ordered assembly structure of LSA further amplified the intermolecular electric fields. Specifically, as the *π‐π* stacking length of LSA increases from 1.7 to 4.4 µm (Figure S12, Supporting Information), achieved by extending the growth time from 4 to 8 h (named LSA‐1 and LSA‐2), the wavelength redshift of UV absorption also increases from 32 to 42 nm. However, with a further increase in growth time to 12 h (named LSA‐3), the π‐π stacking length of LSA decreases to 3.4 µm instead, resulting in a smaller redshift with 36 nm. This phenomenon indicates that the larger the length of LSA, the stronger the *π‐π* stacking effect, which will change the delocalization range of electrons.

The LSA show size‐dependent IEF and photoelectric characteristics, which are the prominent features of the metastable state and will be discussed in the next section. As shown in Figure 3C, LSA‐2 has higher photoinduced ρ with 32.62 µC cm^−2^ compared with that of LSA‐1 (8.52µC cm^−2^) and LSA‐3 (18.02 µC cm^−2^). Similarly, the OCP of LSA‐2 (297 mV) is higher than that of LSA‐1 (230 mV) and LSA‐3 (265 mV, Figure S13, Supporting Information). Thus, the increased multiples of IEF for LSA‐1, LSA‐2, and LSA‐3 are 4.82, 10.69, and 7.52 compared with crystal, respectively. The charge separation efficiency is 30.86%, 70.41%, and 48.74%, respectively (Figure 3D; Figure S14, Supporting Information). Correspondingly, the photoelectric signals are 1.47, 6.95, and 3.37 µA cm^−2^, respectively (Figure S15, Supporting Information). Time‐resolved FL spectroscopy revealed the property of the photo‐generated carriers (Figure 3E; Table S3, Supporting Information). The FL lifetime of PDI crystal, LSA‐1, LSA‐2, and LSA‐3 are 0.59, 0.87, 3.20, and 1.90 ns, respectively. The FL lifetimes of LSA are longer than that of PDI crystal. Moreover, the longer the length of LSA, the longer the lifetime, which indicates that the exciton separation efficiency in LSA is higher and the long‐range transport of photogenerated carriers is more effective. The formation of the LSA can effectively reduce the charge transfer resistance as confirmed by the electrochemical impedance spectroscopy (Figure 3F). The arc radius of LSA is smaller than that of the PDI crystal. The diameter of the arc reflects the charge transfer resistance, and the shorter the arc diameter, the smaller the charge transfer resistance.^[^ ^48^ ^]^ Due to the fact that the stronger *π*‐*π* interaction in LSA increases the electron delocalization, which promotes the separation of photogenerated carriers and inhibits the recombination of photogenerated electrons and holes. The IEF intensity and charge separation efficiency of LSA with growth times of 6 and 10 h were tested (Figures S16 and S17, Supporting Information), and the optimal growth time of LSA was 8 h. At the same time, the IEF and charge separation efficiency of LSA prepared in different ratios of propionic acid and methanol were also tested (Figures S18 and S19, Supporting Information). The results showed that the optimal ratio of propionic acid and methanol was 1: 3 (v/v). It lays the foundation for the subsequent application of LSA.

### 2.4 Customizable IEF with Metastable Nature

**Figure 4:**
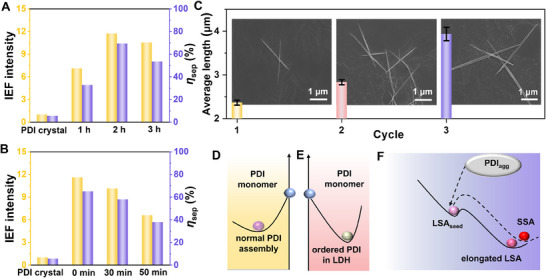
Metastable nature of LSA. The relative IEF intensity and charge separation efficiency of LSA with A) different ultrasonic time, B) changing the timing of addition of CH_3_OH. C) The average length of fresh LSA as a function of cycle number *x*: (*x* = 1, 2, and 3) (insets: corresponding SEM images of fresh LSAs obtained in cycles 1–3, respectively). Schematic illustration for the energy diagrams of D) PDI monomer and normal PDI assembly, E) PDI monomer and PDI‐LDH, and F) the products during the formation of stale LSA.

Due to the thermodynamic control assembly characteristics of conventional SA systems, it is almost impossible to control the size of SA thus realizing control of the IEF. Excitingly, the LSA shows pathway‐dependent IEF and photoelectric characteristics, providing strong evidence for the appearance of the metastable state.^[^ ^49^ ^]^ The change of assembly pathway can be carried out by varying the ultrasonication time and the time of adding the poor solvent CH_3_OH.^[^ ^50^, ^51^, ^52^, ^53^, ^54^ ^]^ With the increase of ultrasonic time, the IEF increased from 7.10 to 11.74 and then decreased to 10.54 compared with PDI crystal (**Figure** **4**A; Figures S20 and S21, Supporting Information). Correspondingly, the change in charge separation efficiency and photocurrent of the LSA is consistent with the change of IEF (Figures S22 and S23, Supporting Information). The electrochemical impedance spectroscopy Nyquist plot results also revealed that the resistance of the LSA first decreased and then increased (Figure S24, Supporting Information). The growth can be directly monitored by SEM images where the length of LSA increased from 2.2 to 4.3 µm and then decreased to 2.9 µm in the ultrasonic time span of 1 to 3 h (Figures S25 and S26, Supporting Information). These indicated that self‐assembly achieves the best results after sonication for a period of time. However, the ultrasonic time is too long, it will produce less living seed and slower autocatalysis,^[^ ^55^, ^56^ ^]^ which affects the elongation of length and *π*‐*π* stacking, subsequently IEF and photoelectric performances. In addition, with increase of the addition time of the poor solvent CH_3_OH from 0 and 30 to 50 min, the length of the LSA decreases from 4.2 to 2.3 µm (Figures S27 and S28, Supporting Information). This is due to a decrease in the rate of LSA nucleation as the CH_3_OH addition time is delayed, as confirmed by the time‐dependent FL spectra (Figures S29–S31, Supporting Information). Thus, the IEF, charge separation efficiency and photocurrent also decrease sequentially (Figure 4B; Figures S32–S35, Supporting Information) and resistance gradually increases (Figure S36, Supporting Information). The pathway‐dependent IEF performance fully demonstrates the metastable characteristics of LSA, which establishes the foundation for the controllable optical and electrical properties of LSA according to requirements.

The growth of LSA is a typical type of living elongation verified by multi‐cycle experiments. With the increase of the cycle number from 1 to 3, the LSA length increased continuously from 2.3 to 3.9 µm as confirmed by SEM images (Figure 4C). The FL emission wavelength of LSA was redshifted from 653 to 665 nm (Figure S37, Supporting Information). The increase in redshift indicates an increase in the assembly degree of LSA, which further confirms the active elongation characteristic of LSA. During the living assembly process, the replicator R can be calculated. The relationship between the logarithm of the apparent assembly rate of LSA (log(d(Abs)/d*t*)) and the logarithm of the seed concentration of LSA_seed_ (log([seeds])) shows that the assembly follows the first‐order reaction rate (Figure S32, Supporting Information and analysis). The results indicated that LSA occurs by exponential replication and living supramolecular assembly.^[^ ^57^, ^58^ ^]^ As the number of cycles increased, the multiples by which IEF increases were 83.4, 99.7, and 119.3 compared with PDI crystal (Figures S38–S41, Supporting Information). The charge separation efficiencies are 43.92%, 55.83%, and 71.71%, respectively (Figure S42, Supporting Information). Thus, the photocurrent increased from 5.04 to 7.97 µA (Figure S43, Supporting Information).

Achieving the exceptional IEF and charge separation efficiency of LSA hinges on the energy trap created by the 2D structure of LDH. This trap overcame the energy barrier associated with the PDI orderly arrangement, thereby ensuring the stability of the released orderly molecular array. The energy transition of PDI molecules through various states is delineated in Figure 4D–F, supported by fluorescence (FL) data rather than theoretical calculations. This reliance on FL data is necessitated by the prohibitive computational demands of calculating energy levels for the successive transformations PDI as the molecule count escalates, outpacing current technological capabilities. The Coulomb force and hydrogen bonds act as the principal driving forces that facilitate PDI incorporation into LDH, implying that the energy level of PDI monomers should exceed that of the ordered PDI within LDH. The higher energy level of PDI monomer compared to PDI‐LDH is corroborated by the redshift in the FL from 580 to 650 nm (Figure S44, Supporting Information). The transition from PDI monomer to ordered PDI in LDH layer overcoming an energy barrier of 23.35 kJ mol^−1^. Consequently, the ordered PDI within LDH is confined in an energy well, maintaining a metastable state. In the transformation process, the energy of elongated LSA (λ_em_ = 654 nm) is lower than that of LSA_seed_ (λ_em_ = 644 nm) (Figure S45, Supporting Information), as confirmed by the red‐shift of FL spectra. Additionally, the intermolecular forces within LSA are bolstered by the confinement effect of LDH, as indicated by the red‐shifted FL and UV‐vis spectra when compared to PDI monomers (Figure S1, Supporting Information). Thus, LDH not only stabilizes the ordered array structure but also amplifies the intermolecular interactions, laying the groundwork for high IEF and photoelectric characteristics.

In the above system, metal ions (Mg^2+^ and Al^3+^) are introduced after the LDH layers are dissolved. In order to eliminate the influence of inorganic ions on the system, we designed a series of comparative samples that have been treated in the same way, including PDI powder and a physical mixture (PDI + LDH). It can be observed that comparative samples can only form rod‐like structures in the SEM images (Figure S46, Supporting Information), which indicates that the introduced metal ions are not the key to promoting the formation of needle shaped LSA.

### 2.5 Application

**Figure 5:**
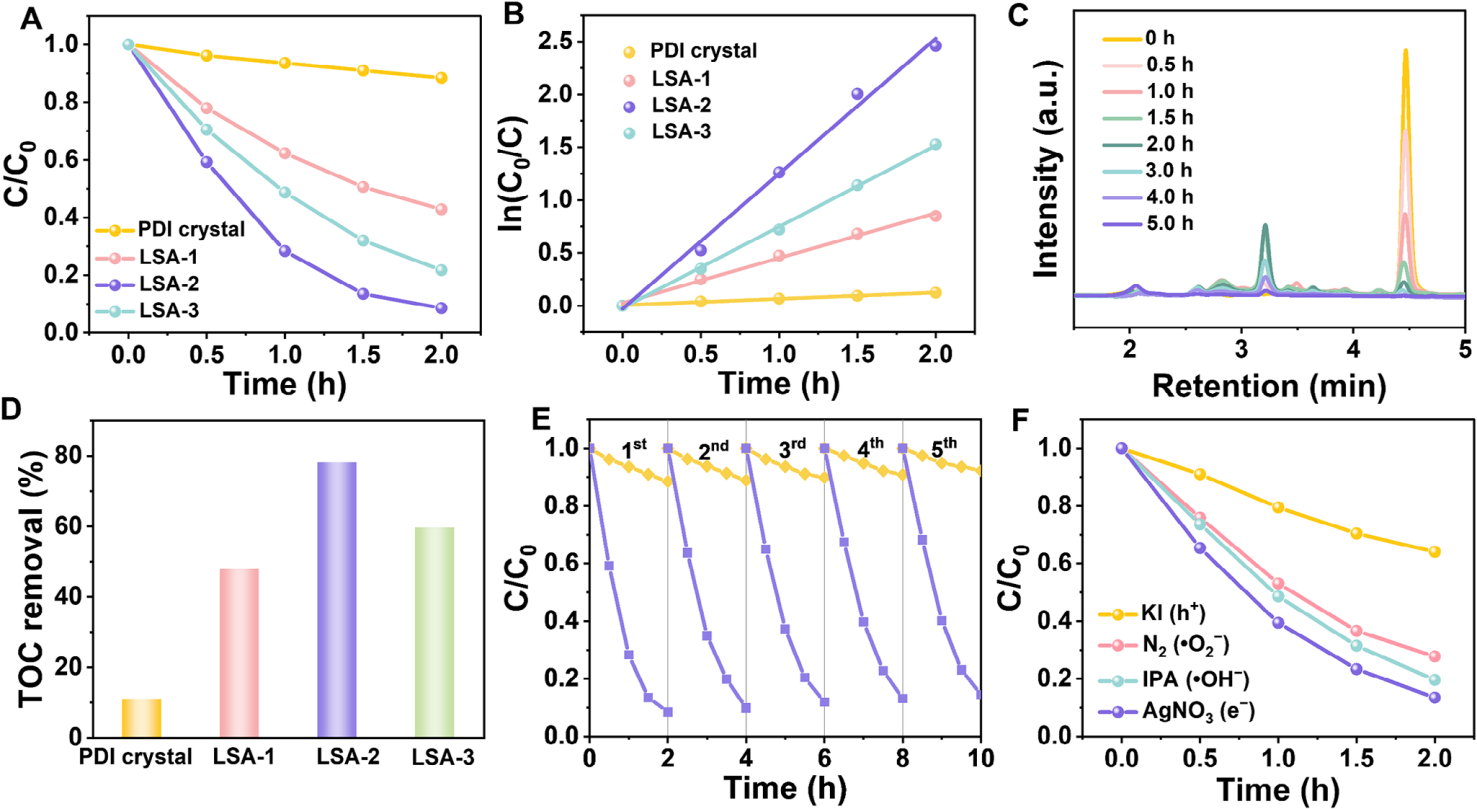
Application of photocatalytic degradation of phenol. A) Phenol photodegradation on PDI crystal, LSA‐1, LSA‐2 and LSA‐3. B) The degradation kinetic curves of phenol by PDI crystal, LSA‐1, LSA‐2, and LSA‐3. C) HPLC chromatograms of phenol degradation process on LSA‐2. D) The TOC removal rate of phenol on PDI crystal, LSA‐1, LSA‐2, and LSA‐3. E) Recycling tests of PDI crystal (yellow line) and LSA‐2 (purple line) for phenol degradation. F) Free radical capture diagram of LSA‐2 degrading phenol under visible light irradiation.

The inherent high efficiency of charge separation in LSA is conducive to the presence of effective electron and hole active species, which suggests that LSA can serve as an effective photocatalyst for the degradation of persistent organic pollutants in the environment. The photocatalytic activity of LSA was evaluated by photocatalytic degradation of phenol under visible light (λ > 420 nm). **Figure** **5**A shows the degradation rate of phenol by PDI crystal and LSA with different length. After 2 h of irradiation, LSA‐2 showed the highest degradation rate of 91.5% for phenol. The degradation process of phenol conformed to the first‐order kinetic equation, and the degradation rate constant (k) was obtained from the slope of the corresponding fitted line (Figure 5B). The degradation rate constant of LSA (1.28 h^−1^) was 21.3 times higher than that of PDI crystal (0.06 h^−1^). Other reported efficient photocatalysts (such as Bi_2_WO_6_ and C_3_N_4_) were also chosen as control samples, with degradation rates of 28% and 43%, and degradation rate constants of 0.16 and 0.29 h^−1^, respectively (Figure S47, Supporting Information). The phenol degradation intermediates were detected by HPLC (Figure 5C). It was evident that the peak of phenol at 4.47 min gradually decreased with irradiation and disappeared at ≈2 h. Under the attack of the LSA photocatalyst, the phenol molecules were decomposed into intermediates, including p‐benzoquinone (3.21 min) and hydroquinone (2.83 min), as confirmed by the intermediate peak increases from 0 to 2 h. Interestingly, as degradation time further increases from 2 to 5 h, the intermediate peak, on the contrary, begins to decrease until it disappears due to phenol thoroughly was decomposed into CO_2_ and H_2_O. Figure 5D shows the TOC removal curve of PDI crystal, LSA‐1, LSA‐2, and LSA‐3. After 5 h of light irradiation, the PDI crystal only mineralized phenol by 10.9%, while the mineralization rate of LSA‐2 was as high as 78.1%, which was 7.2 times higher than that of the NDI crystal, and showed a good ability of deep removal. The mineralization ability of LSA is also better than that of common photocatalysts (Bi_2_WO_6_ and C_3_N_4_) (Figure S48, Supporting Information). The LSA not only degraded phenol to other small molecules, but also mineralized it to CO_2_ and H_2_O. The mineralization ability of LSA is also positively correlated with its size. The LSA catalyst also demonstrated high stability, with negligible reduction in catalytic performance observed after 5 cycles (Figure 5E), whereas PDI crystal showed a decrease in catalytic performance after cycling.

The photocatalytic mechanism was explored by the free radical trapping experiments to demonstrate the types of active species (Figure 5F). Isopropanol (IPA), nitrogen (N_2_), potassium iodide (KI), and silver nitrate (AgNO_3_) were used as trapping agents for ·OH, ·O^2−^, h^+^, and e^−^, respectively. The addition of IPA, AgNO_3,_ and N_2_ has little effect on the degradation rate, while the addition of KI significantly inhibited the degradation rate. These radical trapping experiments indicate that h^+^ is the main active species in the phenol degradation process. The optical properties of PDI crystal and LSA‐2 as a representative were analyzed by UV–vis diffuse reflectance spectroscopy (DRS) (Figure S49, Supporting Information). The absorption edges of PDI crystal and LSA‐2 are 644 and 687 nm, respectively. The light absorption of LSA‐2 in the visible region was significantly enhanced compared to that of PDI crystal. The band gap energy (*E*_g_) values calculated from DRS spectra by Tauc‐Plot are 1.89 and 1.79 eV for PDI crystal and LSA‐2, respectively. Mott–Schottky (MS) test was performed to calculate the flat‐band potential (*E*_fb_). Based on the tangent intercept of the Mott–Schottky curve, the flat‐band potentials of the PDI crystal and the LSA‐2 were −0.36 and −0.21 V, respectively (vs. Ag/AgCl, Figure S50, Supporting Information). The conduction band potential (*E*_CB_) was calculated (details in Supporting Information), where the *E*_CB_ for the PDI crystal and LSA‐2 were −0.25 and −0.10 V (vs. NHE), respectively. Based on the formula of valence band potential (*E*_VB_), the *E*_VB_ of PDI crystal and LSA are 1.64 and 1.89 V (vs. NHE), respectively. Compared with PDI crystal, the *E*_VB_ of LSA‐2 is more positive, which suggests that LSA‐2 has a stronger oxidation ability. In addition, based on the above experimental results, the energy band structures of PDI crystal and LSA‐2 are plotted (Figure S51, Supporting Information). Compared to PDI crystal (1.89 eV), LSA‐2 has a narrower bandgap with 1.79 eV and a longer absorption edge, thus LSA‐2 can absorb more photons, generating electrons (e^−^) and holes (h⁺) in the conduction band and valence band, respectively. Importantly, the strong IEF in LSA‐2, arising from its compact and ordered π‐π stacking architecture, promotes electron delocalization while accelerating the separation and migration of photogenerated carriers, thereby achieving significantly improved photocatalytic efficiency compared to conventional PDI systems.

### 2.6 Universality

**Figure 6:**
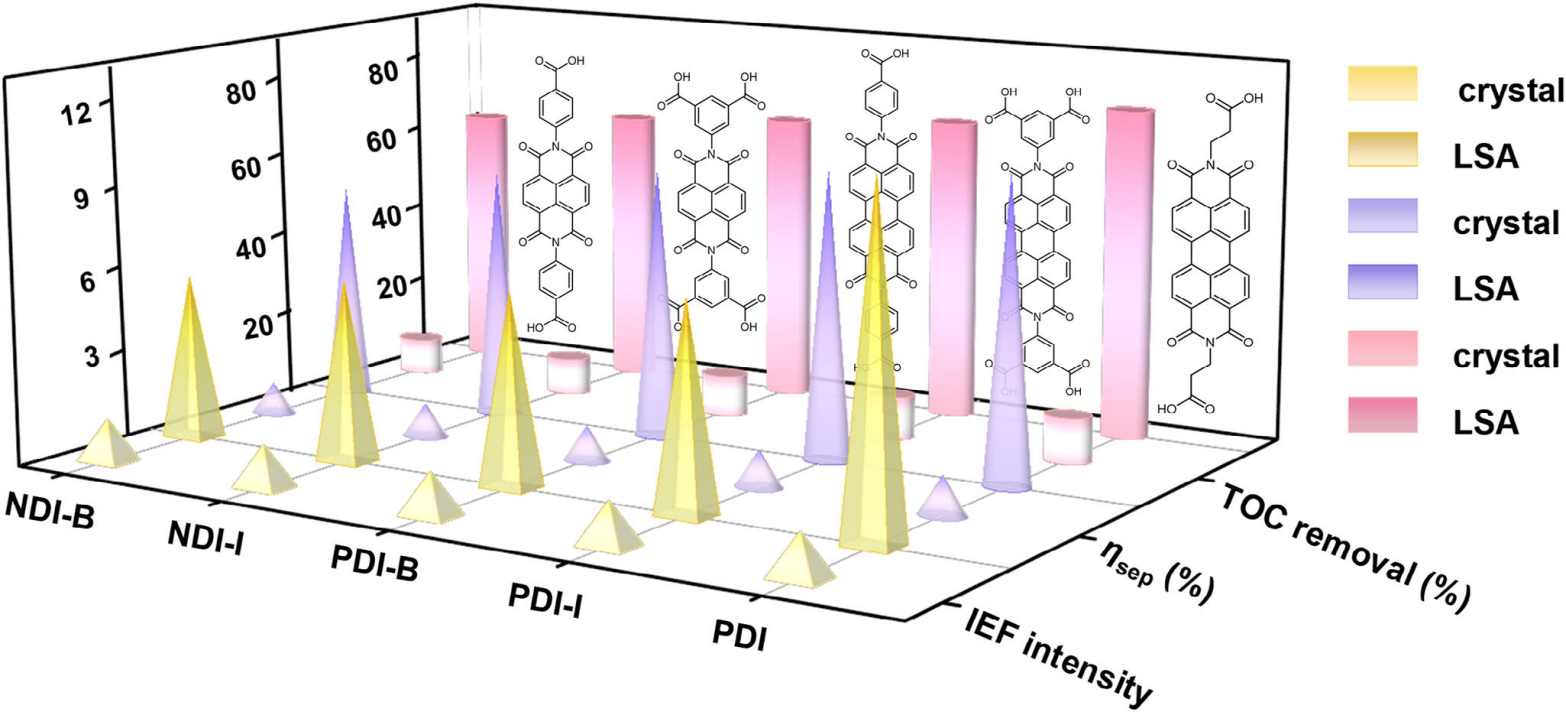
Test of universality. A) Various molecules were chosen in our proposed method. All chemical structures are obtained in ChemDraw. B) IEF intensity, charge separation efficiency, and TOC removal rate of crystals and corresponding LSAs.

In order to verify the generality of our proposed strategy to enhance the IEF of organic molecules by preparing LSA via a 2D confinement space, we have chosen four additional organic molecules to be ordered in the confinement space of the LDH (**Figure** **6**; Figure S52, Supporting Information), including N,N'‐bis(4‐carboxyphenyl)naphthalene‐1,4,5,8‐tetracarboxylic diamide, NDI‐B; N,N'‐bis(5‐isophthalato)naphthalene‐1,4,5,8‐tetracarboxylic diamide, NDI‐I; N,N'‐bis(4‐carboxyphenyl)perylene‐3,4,9,10‐tetracarboxylic diamide, PDI‐B; N,N'‐bis(5‐isophthalato)perylene‐3,4,9,10‐tetracarboxylic diamide, PDI‐I. The corresponding assemblies were obtained by further performing the same assembly process as that of the PDI (Figures S53–S59, Supporting Information), which are named as LSA_NDI‐B_, LSA_NDI‐I_, LSA_PDI‐B_, LSA_PDI‐I_, respectively. As testified by FL polarization spectra (Figures S60–S63, Supporting Information), all of the LSAs show a higher anisotropic value compared to the corresponding crystals. The confinement‐mediated LSA consistently produces tighter π‐π stacking compared to the crystal molecule (Figure S64 and Table S4, Supporting Information). All as‐prepared LSA have significantly enhanced IEF and charge separation efficiency compared to the corresponding crystals. Based on the experimental results, the basic principles are summarized as follows: (1) the monomeric conjugation degree is directly proportional to the IEF. For example, the IEF of LSA composed of by perylene diimide (LSA_PDI‐B_, LSA_PDI‐I_, and LSA_PDI_) is larger than that of naphthalene diimide (LSA_NDI‐B_ and LSA_NDI‐I_) because larger conjugated structure is more conducive to electron transportation; (2) the steric hindrance of the molecule is inversely proportional to the IEF. For example, the steric hindrance of PDI‐B and PDI‐I is greater PDI, leading to the corresponding IEF of LSA_PDI‐B_ and LSA_PDI‐I_ is smaller than that of LSA_PDI_. This is because that the terminal substitutions increase the *π*‐*π* distance of the benzene ring. As shown in the XRD patterns (Figure S65, Supporting Information), the angle of *π*‐*π* diffraction peak for LSA_PDI_ and LSA_PDI‐B_ (2*θ* = 28.80° and 28.51°) is larger than LSA_PDI‐I_ of 28.18°. Therefore, the distances between adjacent molecules of LSA_PDI_ (*d* = 3.098 Å) and LSA_PDI‐B_ (*d* = 3.128 Å) are smaller than LSA_PDI‐I_ of 3.165 Å; (3) the number of ─COOH groups is directly proportional to the IEF, which affects the formation of H‐bond. For example, PDI‐I has more ─COOH than PDI‐B, which is beneficial for obtaining a large hydrogen bond network. As confirmed by FT‐IR spectra, the increased intensity of OH in LSA_PDI‐I_ at 3425 cm^−1^ and shifted to the lower wavenumber of C ═ O for compared with LSA_PDI‐B_ (Figure S66, Supporting Information). In summary, the ranking order of the influencing factors of IEF is conjugation degree > steric hindrance > the number of ─COOH (Figures S67–S78, Supporting Information). The relationship between charge separation efficiency and TOC removal rate of LSAs with molecular structure also exhibits the same trend as IEF. Time‐resolved fluorescence tests (Figure S79 and Table S5, Supporting Information) and electrochemical resistance tests (Figure S80, Supporting Information) further confirmed that LSA is beneficial to separation of photogenerated carriers and inhibits the recombination of photogenerated electrons and holes.

## 3 Conclusion

With the benefit of LDH confinement and LSA technology, we propose a method to enhance IEF, where the highest relative IEF intensity can reach 11.8 times, representing the current state‐of‐the‐art research advancements. Benefiting from its strong intrinsic electric field (IEF), the LSA assembly achieves a remarkable charge separation efficiency of 71.52%, which synergistically accelerates carrier transport kinetics. The LSA‐2 photocatalyst achieves a phenol degradation rate constant of 1.28 h^−1^, which is 21 times higher than that of crystalline PDI and exceeds the performance of benchmark catalysts Bi_2_WO_6_ (0.29 h^−1^) and C₃N₄ (0.16 h^−1^) by 4–8 fold. Simultaneously, it attains 78.1% total organic carbon (TOC) mineralization efficiency, representing a 7.2‐fold improvement over the PDI reference system (10.9% TOC removal). The energy trap of LDH overcomes the energy barrier of molecular orderly arrangement, not only reducing the distance between adjacent molecules in LSA compared to that in the corresponding crystal, but also endowing the ordered array LSA with the ability to live growth. The longer‐range ordered *π*‐*π* stacking induced the polarization and amplified the intermolecular dipole‐induced IEF, which facilitates charge separation and transfer, resulting in LSA with a higher photocatalytic activity. We assert that our system is not unique in this regard. Amplified IEF and charge separation efficiency can be facilitated by a variety of molecules, potentially opening up new avenues for designing functional materials. It allows researchers to explore novel applications and paves the way for advancements in fields like material science, efficient photocatalysis, and chemical synthesis.

## 4 Experimental Section

### Chemicals

MgCl_2_·6H_2_O, AlCl_3_·6H_2_O, NaOH, CH_3_OH were purchased from Beijing Chemical Reagent Company. Propionic acid, methanol, perylene‐3,4,9,10‐tetracarboxylic diimide (PDI), Beta‐alanine, imidazole, phenol, and KI were obtained from J&K Chemical. Isopropanol was purchased from Aladdin Holdings Group Co., Ltd. AgNO_3_ was purchased from Sinopharm Chemical Reagent Co., Ltd. In this work, all reagents were used as received without further purification. All solutions were prepared with decarbonated deionized water.

### Synthesis of PDI‐LDHs

Mg_3_Al‐Cl‐LDH was prepared. First, NaCl solution (1.00 m) of 50.0 mL was added into a 500 mL four‐necked flask. 50.0 mL of salt solution containing 3.75 mmol MgCl_2_·6H_2_O and 1.25 mmol AlCl_3_·6H_2_O was marked as solution A. 50.0 mL NaOH solution (1.00 m) was marked as solution B. Both of them were added dropwise into the flask simultaneously under vigorous stirring. The pH of the suspension was maintained at 8.00 throughout the process. The mixture was kept stirring and aged at room temperature under N_2_ atmosphere for 24 h. Wash with decarbonated water to give a colloid of Mg_3_Al‐Cl‐LDH. Ready for use after quantification.

PDI‐LDHs (LDH) were prepared via the ion‐exchange method. 0.313 g Mg_3_Al‐Cl‐LDH were added into PDI solution (10.0 mM, pH = 9.0) at 80 °C under N_2_ atmosphere, respectively. PDI‐LDHs were obtained by ion‐exchange for 72 h. The products were washed with deionized water and CH_3_OH respectively for three times and re‐dispersed in CH_3_OH as stocking solution (30.0 g L^−1^) for further use. All water used in the process was treated with distillation.

### Synthesis of LSA

The LSA was prepared based on the previous work.^[^ ^59^ ^]^ The long *π*‐*π* PDI arrays could be released from as‐prepared PDI‐LDHs (3.00 mg) in 800 µL propionic acid/CH_3_OH (1:3 v/v) solvent. The released product was sonicated for 1 h to expose more active sites, followed by an 8 h growth period to achieve a stable energy state for LSA. The LSAs grown for 4, 8, and 12 h were designated as LSA‐1, LSA‐2, and LSA‐3, respectively. A mixture of ice and water was used to maintain a constant temperature of 0 °C during the ultrasound process.

### Reversibility of LSA

The LSA was first prepared by dissolving 3.00 mg PDI‐LDH into 800 µL propionic acid/CH_3_OH (1:3 v/v). First, the disassembly of LSA happened after adding 100 µL good solvent into 800 µL propionic acid/CH_3_OH (1:3 v/v). After that, the disassembled LSA could re‐assemble after adding 300 µL poor solvent (CH_3_OH) into 900 µL the above solution.

### Tuning the Size of LSA Via Ultrasound Time

The effect of ultrasound time on the size of LSA could be seen in SEM images by prolonging ultrasound time (1, 2, and 3 h). During ultrasound, the mixture of ice and water was used to keep a constant temperature at 0 °C.

### Tuning the Size of LSA Via the Addition Time of CH_3_OH

By changing the time when the CH_3_OH was added during the self‐assembly process, different kinetic assembly of the living seed was obtained. The specific experimental steps are as follows: Condition (1) 0 min (300 µL); Condition (2) 0 min (100 µL) and 30 min (200 µL); Condition (3) 0 min (100 µL), 50 min (200 µL). By changing the CH_3_OH addition time (0, 30, and 50 min), different sizes of LSA which could be seen in SEM images can be obtained.

### Living Nature of LSA (Multi‐Cycle Experiments)

PDI was dispersed in a propionic acid/ CH_3_OH solvent mixture (1: 3 v/v) to obtain a supersaturated solution, named PDI_agg_. The product of cycle 1 was prepared by mixing equal volumes of living seed (4.00 mm) and PDI_agg_ (2.50 mm) with ultrasound for 1 h. Cycle 2 and cycle 3 products were duplicated by adding PDI_agg_ (2.50 mm) further to the previous cycle product (1:1 v/v) to repeat LSA. During ultrasound, the mixture of ice and water was used to maintain a constant temperature of 0 °C.

### Testing of Charge Separation Efficiency

The charge separation efficiency (*η*_sep_) was investigated by adding a fast electron scavenger (H_2_O_2_) to the electrolyte solution. According to the ratio of photocurrents (*J*) detected in H_2_O and H_2_O_2_ electrolytes, the charge separation efficiency in the surface *η*_sep_ could be determined as follows:

(2) $$\eta_{\mathrm{sep}}=J_{H_{2}O}/J_{H_{2}O_{2}}$$

where $J_{H_{2}O}$ and $J_{H_{2}O_{2}}$ were the photocurrent density in the Na_2_SO_4_ aqueous solution without and with adding H_2_O_2_, respectively.

### Evaluation of Photocatalytic Performance

25 mg of photocatalyst (LSA) was dispersed in 50 mL of phenol solution (5 ppm). The solution was stirred in a dark environment for 30 min to achieve the equilibrium of adsorption and desorption. Then, the photocatalytic reaction was started under the irradiation of a 300 W xenon lamp (PLS‐SXE300E, Beijing Perfectlight) with a 420 nm cut‐off filter. 2.5 mL of the reaction solution was taken out every 30 min, then the solid photocatalyst was removed by centrifugation, and the upper suspension was further filtered with a microporous filter membrane (diameter 0.45 µm). The concentration of phenol at different reaction times was determined by UV–Vis spectrophotometer. The TOC of the reacted solution was tested by using a total organic carbon analyzer.

### Testing of IEF

The intensity of the IEF is calculated by the following formula:

(3) $$F_{S}={\left(\frac{-2V_{s}\rho }{\varepsilon \varepsilon_{0}}\right)}^{1/2}$$

where *F*_s_ is the magnitude of the internal electric field, *V*_s_ was the surface photovoltage, *ρ* was the surface charged density, Ɛ for low frequency dielectric constant, Ɛ_0_ for the permittivity of free space. Since the Ɛ and Ɛ_0_ are two constants, the *F*_s_ mainly decided by the surface voltage and charge density. The surface charge density could be obtained by integrating the measured transient photocurrent density minus the steady‐state value of photocurrent with respect to time, which was proportional to the amount of positive charge accumulated on the surface. The surface photovoltage is the open‐circuit potential difference before and after turning on the light.

### Model Construction

For computational cost‐effectiveness, the assembly contained 4 PDI molecules. Furthermore, the effect of the number of PDI molecules is investigated by building an assembly containing 4 PDI molecules.

### Computational Details

In order to obtain the packing mode of molecules, the geometry of an isolated molecule was optimized at the B3LYP/def2SVP level using the Gaussian16 software package. Electrostatic potential (ESP) derived atomic point charges were determined by fitting the molecular ESP at the atomic centers at the same level using Multiwfn. Four probable space groups were restricted in each space group when structure prediction was carried out using the polymorph module of Materials Studio 2020. The crystal structure that best matched the XRD experiment was selected and the resulting structure was then optimized using the CP2K/Quickstep software. Single point energy calculations using ORCA‐5.0.4 at the B3LYP/def2SVPD level were conducted to determine the dipole moment.

### Apparatus and Characterization

XRD powders were measured on an XRD‐6000 (Shimadzu, Japan), with a scan rate of 10° min^−1^ and a scan scope ranging from 3 to 90° for 2*θ* angle. SEM images were characterized by HT7800 (Hitachi, Japan). TEM images were obtained from HT7700 (Htachi, Japan). ICP‐MS results were obtained by using an iCAP6300 Radial (Thermo Fisher Scientific, USA). The elemental analysis was tested on vairo EL CUBE (Elementar, Germany). The fluorescence spectra were performed on an F‐7000 fluorescence spectrophotometer (Hitachi, Japan). UV–vis spectra were recorded on UV‐3900H (Hitachi, Japan). Polarized FL profiles and anisotropic value (*r*) were tested on FLS980 (Edinburgh, UK). FTIR spectra were obtained by a Nicolet 6700 FTIR spectrometer (Thermo, America). The lifetimes were measured by an Edinburgh FLS 980 Steady State Spectrometer. Electrochemical measurements were performed on a CHI660E electrochemical workstation, using a standard three‐electrode cell with a working electrode, a Pt counter electrode, and Ag/AgCl reference electrode. The electorate was 0.1 m Na_2_SO_4_ solution. Photocatalytic solutions of different reaction times were tested using HPLC (Agilent 1200, USA) equipped with a C18 reversed‐phase column. The TOC was measured by a total organic carbon analyzer (TOC‐L CPH, Shimadu, Japan).

## Conflict of Interest

The authors declare no conflict of interest.

## Supporting information



Supporting Information

## Data Availability

The data that support the findings of this study are available from the corresponding author upon reasonable request.
