# Supplementary material for: Confinement‐Induced Metastable Supramolecular Assembly: Dynamic Built‐in Electric Field Driving Deep Mineralization of Organic Pollutants
Source: Adv Sci (Weinh). 2025 Jun 5;12(32):e06797. doi: 10.1002/advs.202506797 (PMC12407308; doi:10.1002/advs.202506797)
Supplement: Supplementary file 1 — Supporting Information [file ADVS-12-e06797-s001.docx]

Supporting Information

Confinement-Induced Metastable Supramolecular Assembly: Dynamic Built-in Electric Field Driving Deep Mineralization of Organic Pollutants

Xiaoqing Cao, Kaixiang Liang, Feifei Peng, Xianggui Kong, and Wenying Shi*

State Key Laboratory of Chemical Resource Engineering, Beijing University of Chemical Technology, 15 Beisanhuan East Road, P. Box 98, 100029, Beijing (P. R. China).

E-mail: shiwy@mail.buct.edu.cn


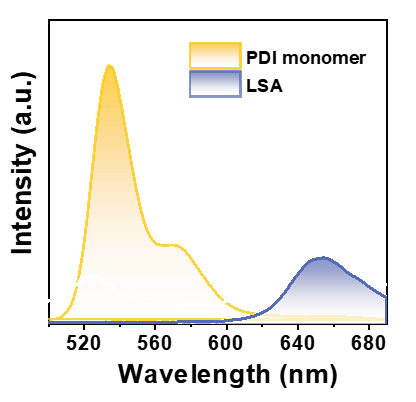


**Figure S1**. FL emission spectra of PDI monomer and LSA.


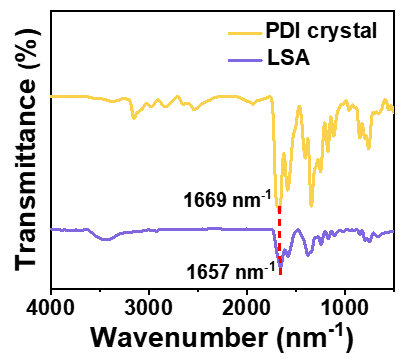


**Figure S2.** The FT-IR spectra of (a) PDI crystal and (b) LSA.


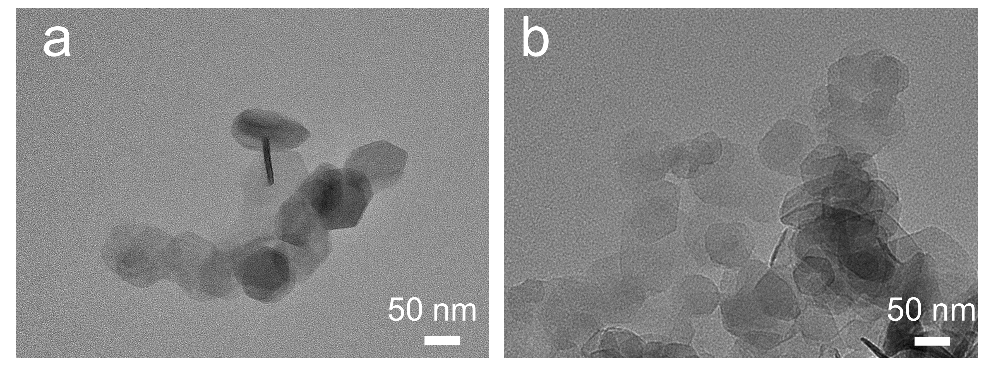


**Figure S3.** TEM images of (a) MgAl-LDH and (b) PDI-LDH.


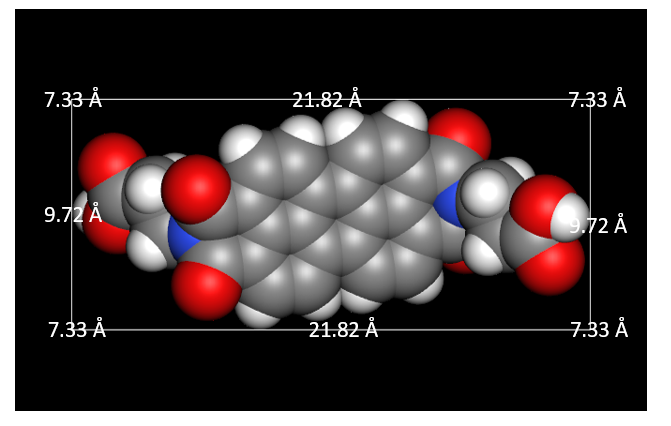


**Figure S4.** The size of the PDI molecule.


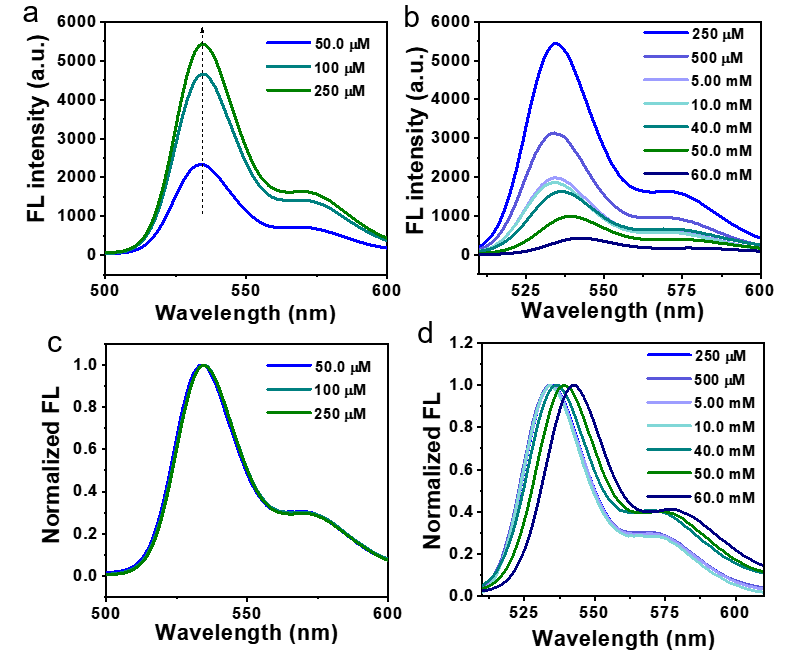


**Figure S5.** FL spectra of (a) 5.00−25.0 μM PDI powder and (b) 0.025−2.50 mM PDI powder dispersed in propionic acid/CH_3_OH (1:3 v/v). (c−d) Normalized FL spectra of (a−b).


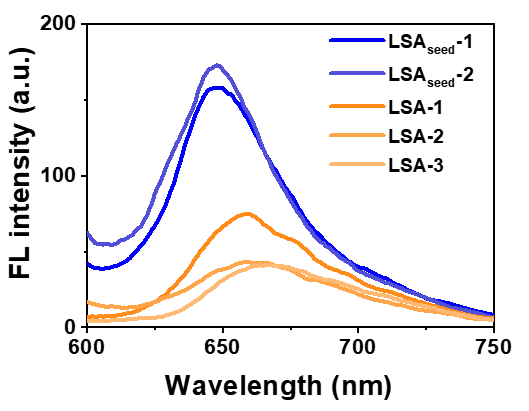


**Figure S6.** FL spectra of the reversible disassembly (LSA_seed_-1 and LSA_seed_-2) and re-assembly (LSA-1, LSA-2 and LSA-3) of LSA.


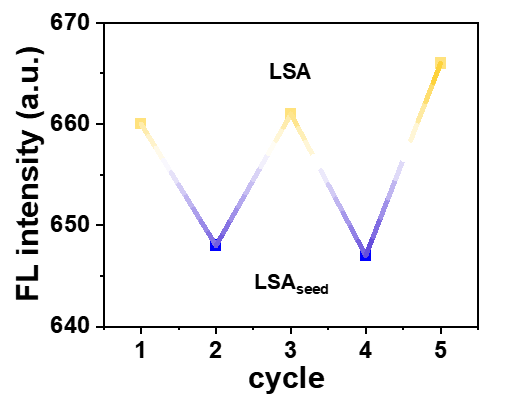


**Figure S7.** FL spectra of the reversible disassembly (λem = 645−650 nm) and re-assembly (λem = 665 nm) of LSA.


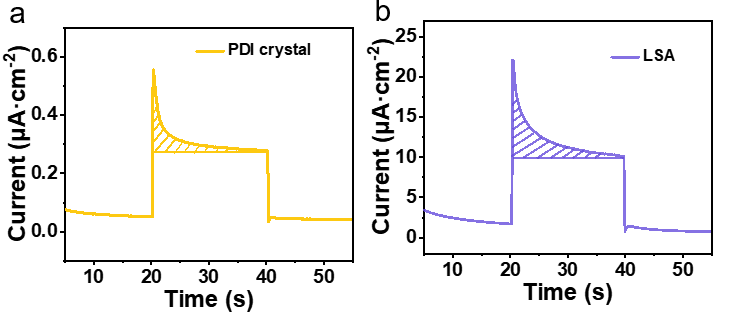


**Figure S8.** The photocurrent density of (a) PDI crystal and (b) LSA.


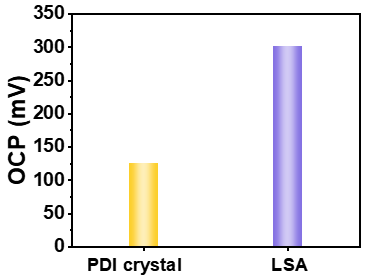


**Figure S9.** The open circuit potential of PDI crystal and LSA.


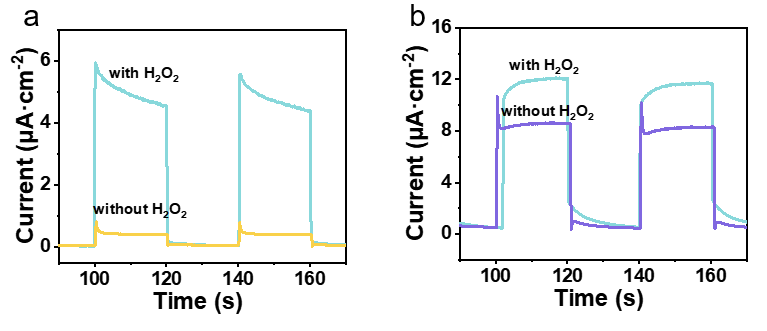


**Figure S10.** The transient photocurrent density with and without adding H_2_O_2_ of (a) PDI crystal and (b) LSA.


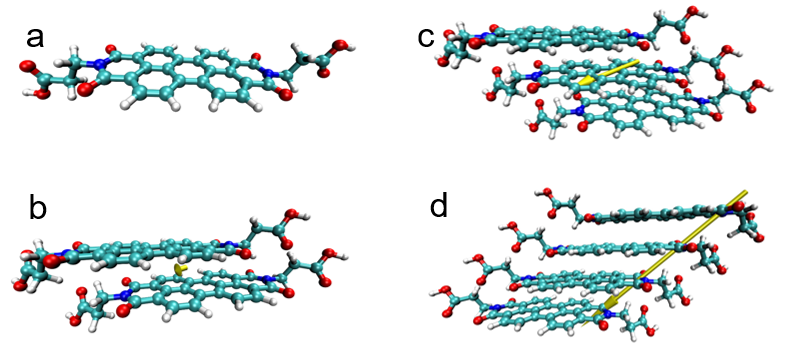


**Figure S11.** Dipole moment of (a) isolated molecule, (b) dimer, (c) trimer and (d) tetramer. The arrow indicates the direction of the dipole moment, from the center of positive charge to the center of negative charge.


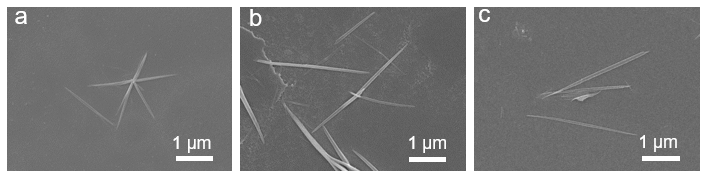


**Figure S12.** SEM images of living chiral units elongated for different time: (a) 4 h, (b) 8 h and (c) 12 h.


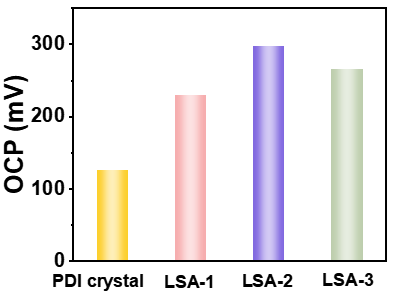


**Figure S13.** The open circuit potential of PDI crystal, LSA-1, LSA-2 and LSA-3.


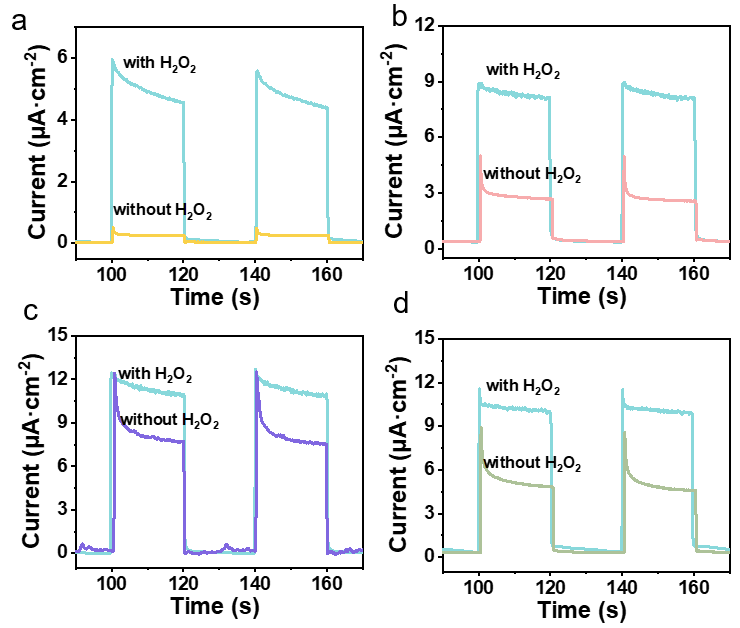


**Figure S14.** The transient photocurrent density with and without adding H_2_O_2_ of (a) PDI crystal, (b) LSA-1, (c) LSA-2 and (d) LSA-3.


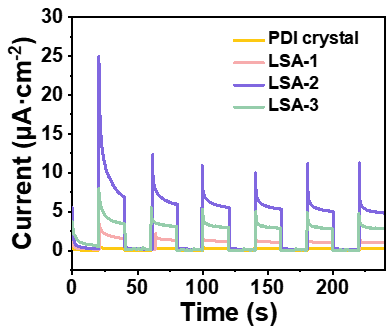


**Figure S15.** The photocurrent density of PDI crystal, LSA-1, LSA-2 and LSA-3.


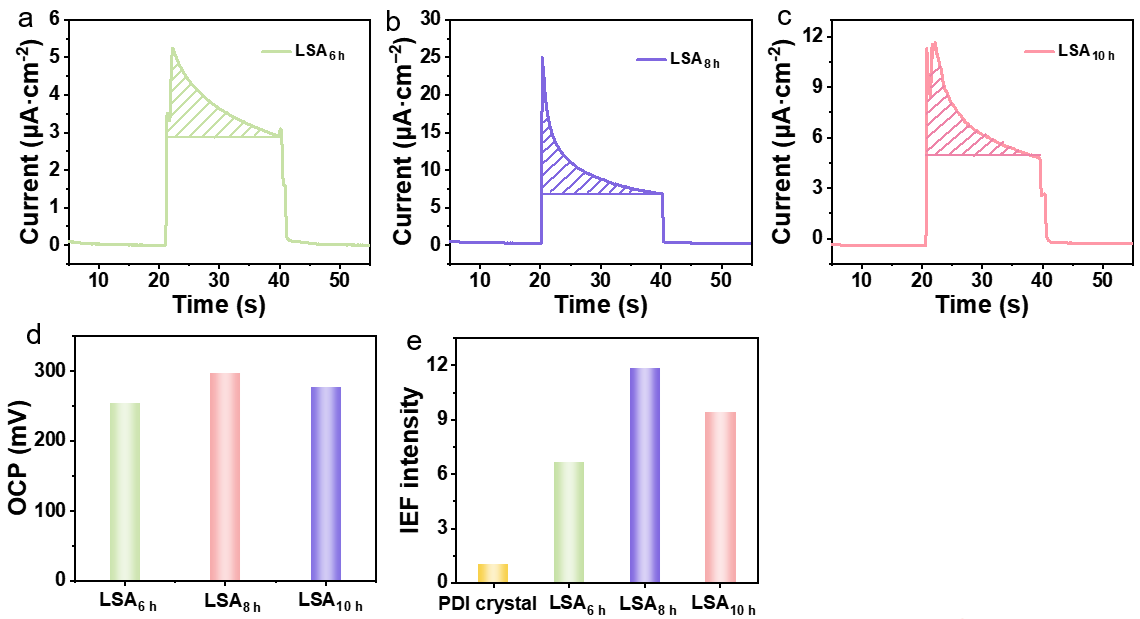


**Figure S16.** The photocurrent density of (a) LSA_6 h_, (b) LSA_8 h_ and (c) LSA_10 h_. (d) The open circuit potential of LSA_6 h_, LSA_8 h_ and LSA_10 h_. (e) The IEF intensity of LSA_6 h_, LSA_8 h_ and LSA_10 h_.


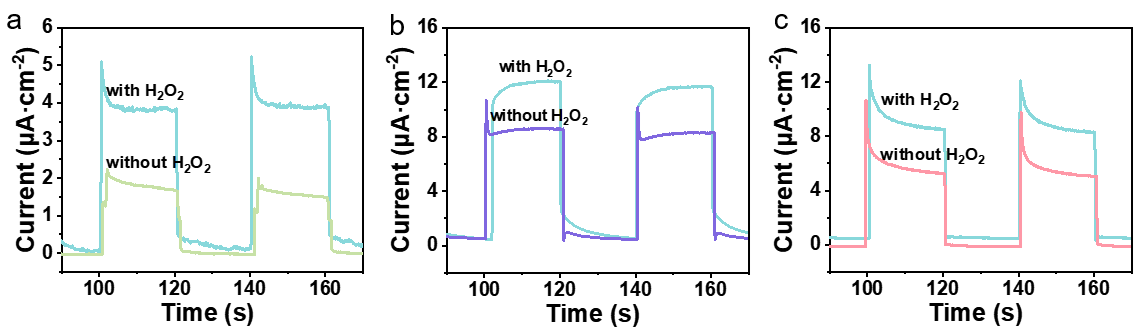


**Figure S17.** The transient photocurrent density with and without adding H_2_O_2_ of (a) LSA_6 h_, (b) LSA_8 h_ and (c) LSA_10 h_.


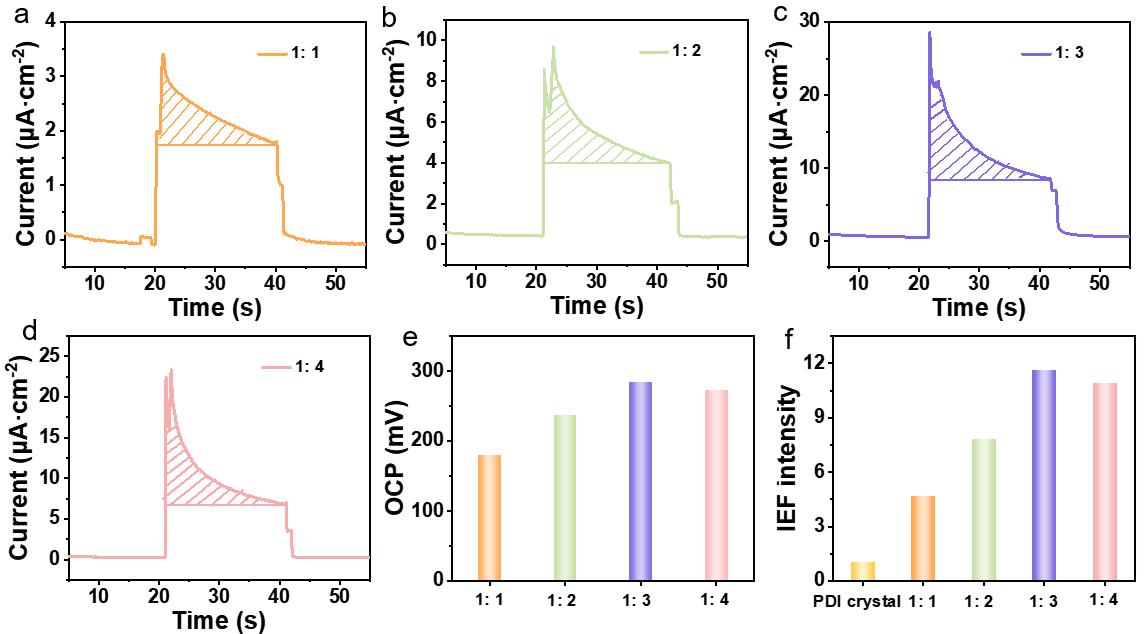


**Figure S18.** The photocurrent density of propionic acid and methanol in different ratios: (a) 1: 1, (b) 1: 2, (c) 1: 3 and (d) 1: 4. (e) The open circuit potential of propionic acid and methanol in different ratios. (f) The IEF intensity of propionic acid and methanol in different ratios.


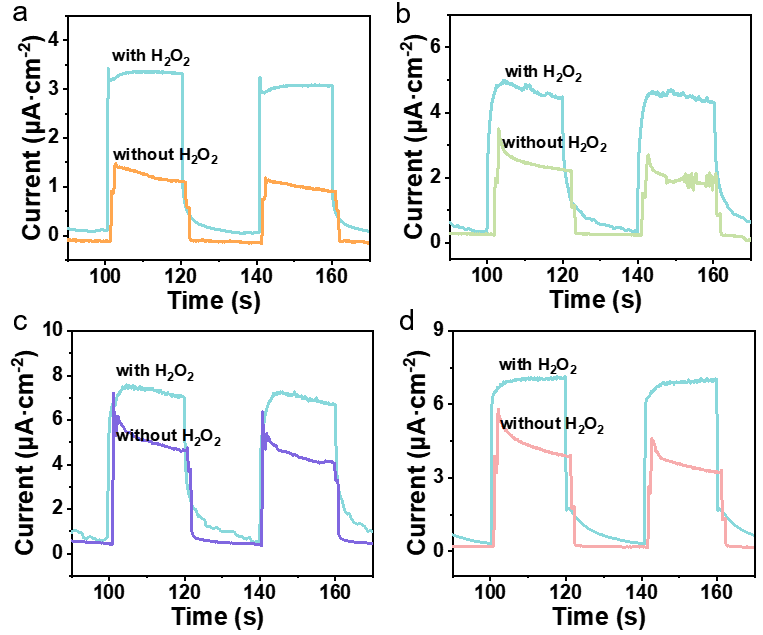


**Figure S19.** The transient photocurrent density with and without adding H_2_O_2_ of propionic acid and methanol in different ratios: (a) 1: 1, (b) 1: 2, (c) 1: 3 and (d) 1: 4.


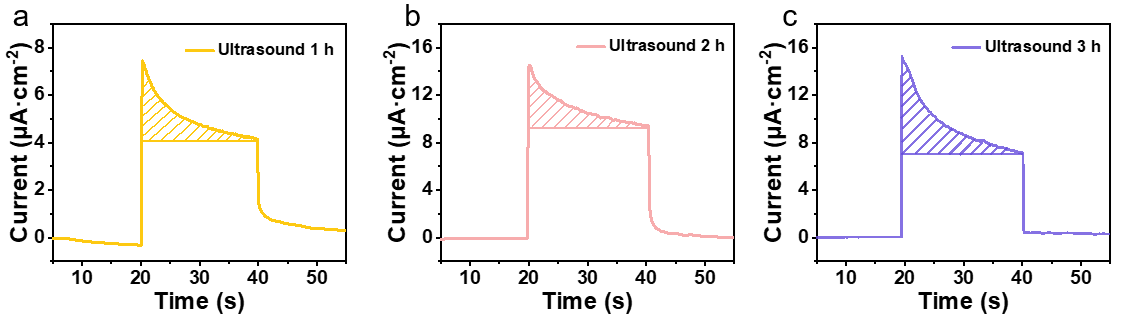


**Figure S20.** The photocurrent density of LSA ultrasound 1 h, 2 h and 3 h. The photogenerated *ρ* varied from 16.98, 39.96 to 35.78 μC/cm^2^ with increase in the ultrasonic time from 1 to 3 h.


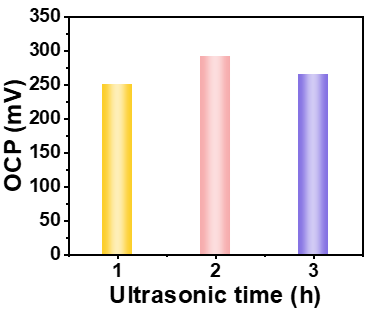


**Figure S21.** The open circuit potential of LSA ultrasound 1 h, 2 h and 3 h. The OCP varied from 251, 292 to 263 mV with increase in the ultrasonic time from 1 to 3 h.


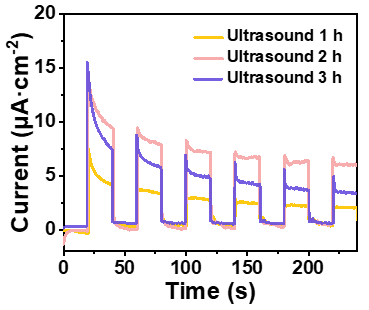


**Figure S22.** The photocurrent density of LSA ultrasound 1 h, 2 h and 3 h.


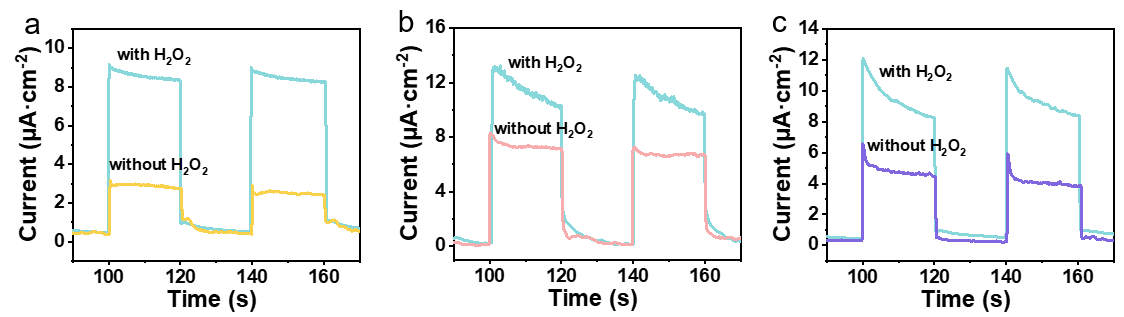


**Figure S23.** The transient photocurrent density with and without adding H_2_O_2_ of LSA ultrasound (a) 1 h, (b) 2 h and (c) 3 h.


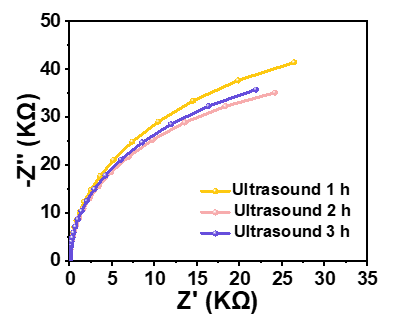


**Figure S24.** Electrochemical impedance spectra of LSA ultrasound 1 h, 2 h and 3 h.


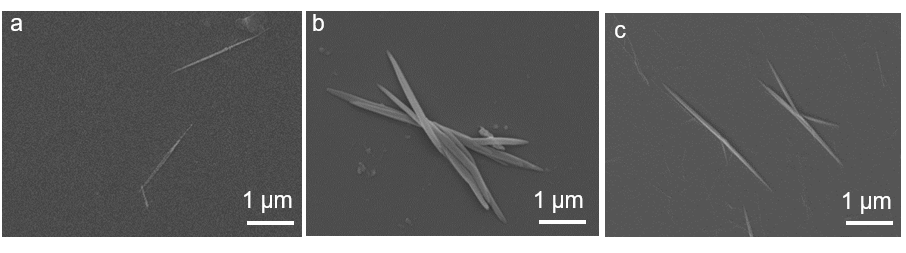


**Figure S25.** SEM images of LSA ultrasound (a) 1 h, (b) 2 h and (c) 3 h.


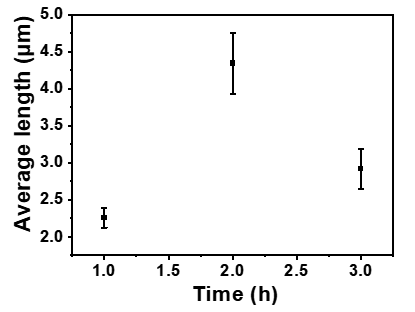


**Figure S26.** The average length of LSA ultrasound 1 h, 2 h and 3 h.


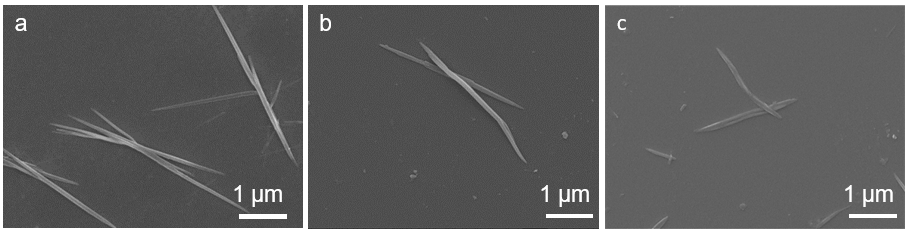


**Figure S27.** SEM images of LSA *via* changing the timing of addition of CH_3_OH: (a) 0 min, (b) 30 min and (c) 50 min.


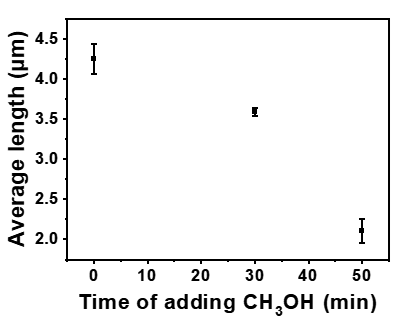


**Figure S28.** The average length of LSA *via* changing the timing of addition of CH_3_OH: 0 min, 30 min and 50 min.


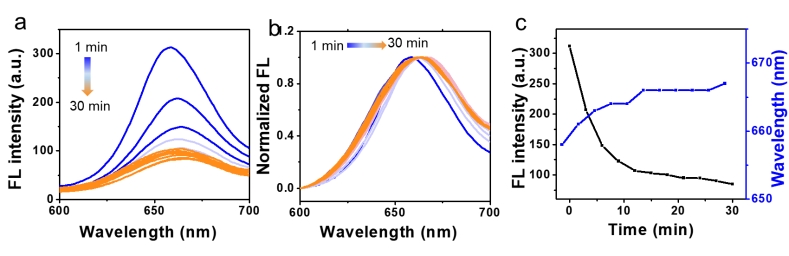


**Figure S29.** (a) FL spectra of metastable LSA *via* changing the timing of addition of CH_3_OH: 1−30 min. (b) Normalized FL spectra of (a). (c) Growht time-dependent FL intensity and wavelength of (a).


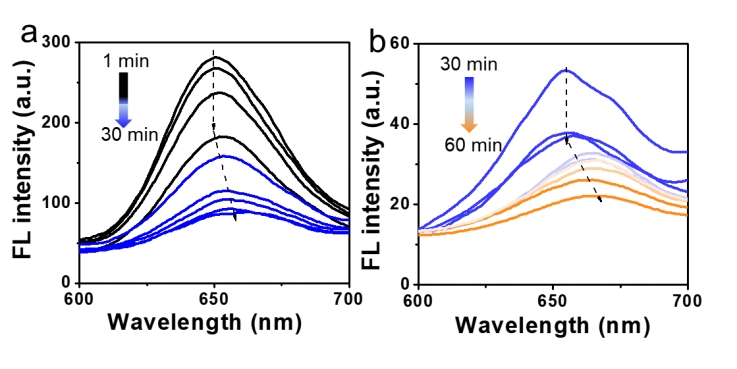


**Figure S30.** FL spectra of metastable LSA *via* changing the timing of addition of CH_3_OH: (a) 1−30 min and (b) 30−60 min.


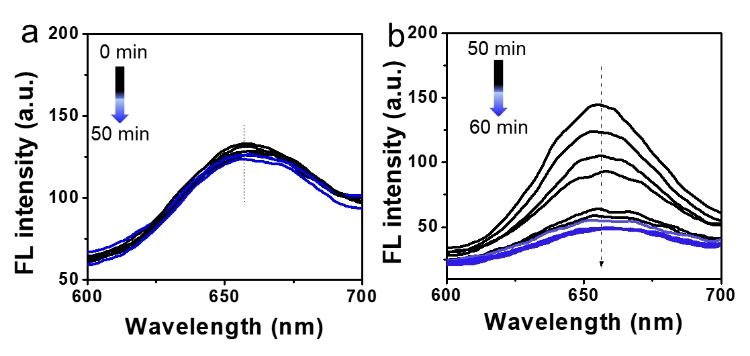


**Figure S31.** FL spectra of metastable LSA *via* changing the timing of addition of CH_3_OH: (a) 1−50 min and (b) 50−60 min.


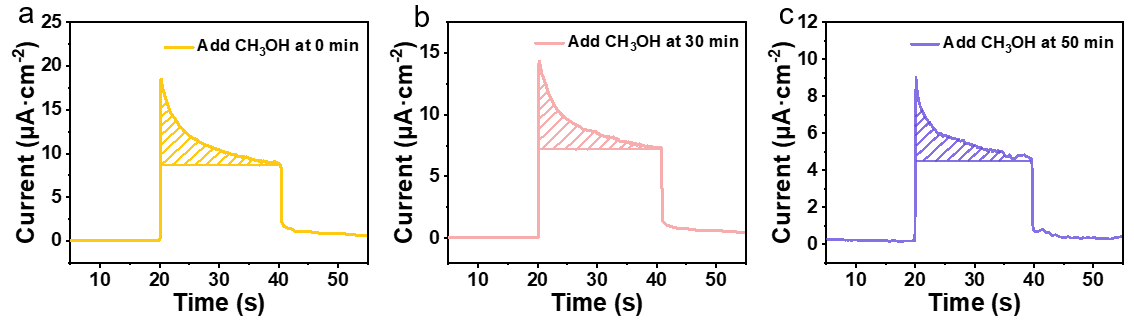


**Figure S32.** The photocurrent density of LSA *via* changing the timing of addition of CH_3_OH: (a) 0 min, (b) 30 min and (c) 50 min.


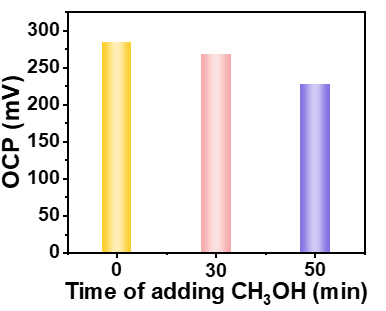


**Figure S33.** The open circuit potential of LSA *via* changing the timing of addition of CH_3_OH: (a) 0 min, (b) 30 min and (c) 50 min.


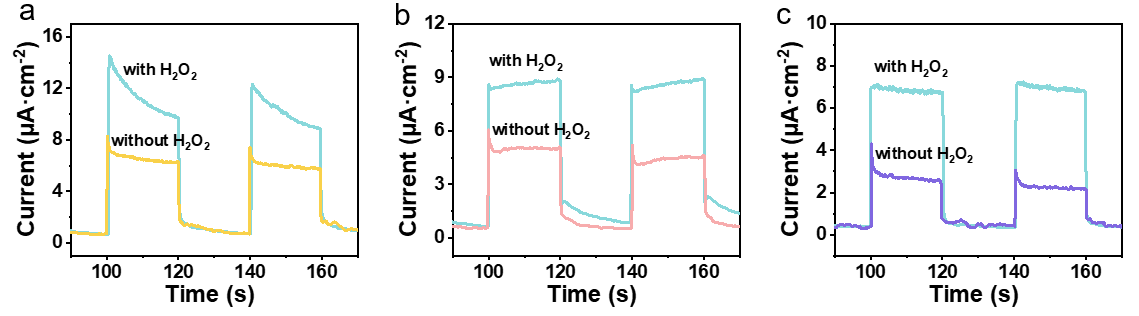


**Figure S34.** The transient photocurrent density with and without adding H_2_O_2_ of LSA *via* changing the timing of addition of CH_3_OH: (a) 0 min, (b) 30 min and (c) 50 min.


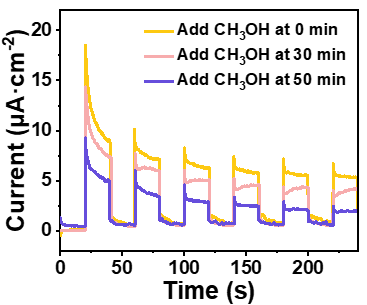


**Figure S35.** The photocurrent responses measured over the ITO of LSA *via* changing the timing of addition of CH_3_OH: (a) 0 min, (b) 30 min and (c) 50 min.


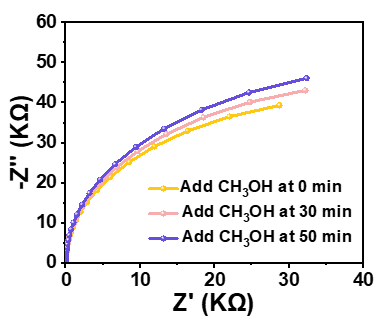


**Figure S36.** Electrochemical impedance spectra of LSA *via* changing the timing of addition of CH_3_OH: (a) 0 min, (b) 30 min and (c) 50 min.

**
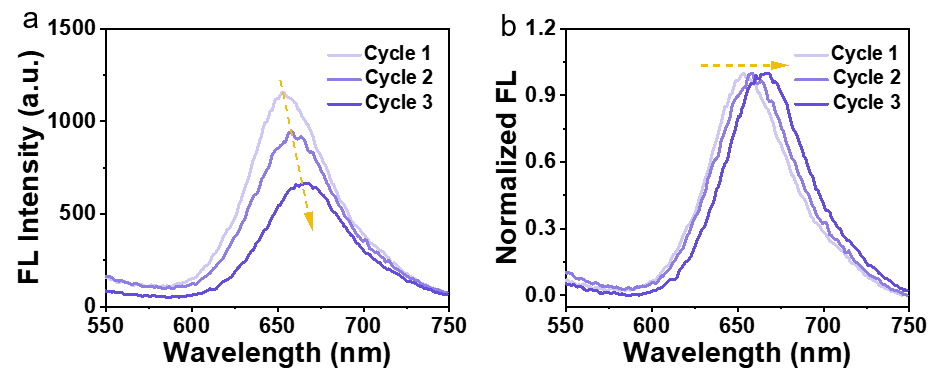
**

**Figure S37.** (a) FL spectra and (b) normalized FL spectra of resulting assembly obtained in Cycle 1–3.


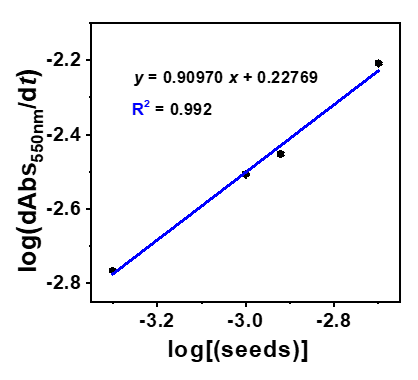


**Figure S38.** Log–log plot of the rate of increased absorbance at 550 nm as a function of living seed concentration. In the living assembly process, the replicator R can be calculated by Equation (1). Where F is a food molecule, *k*_R_ is the rate constant, *f* and *r* are the order of the replication process in food and replicator, respectively.

$\log\frac{d[R]}{dt}=logK_{R}+f\log\left[ F \right]+rlog[R]$ (1)


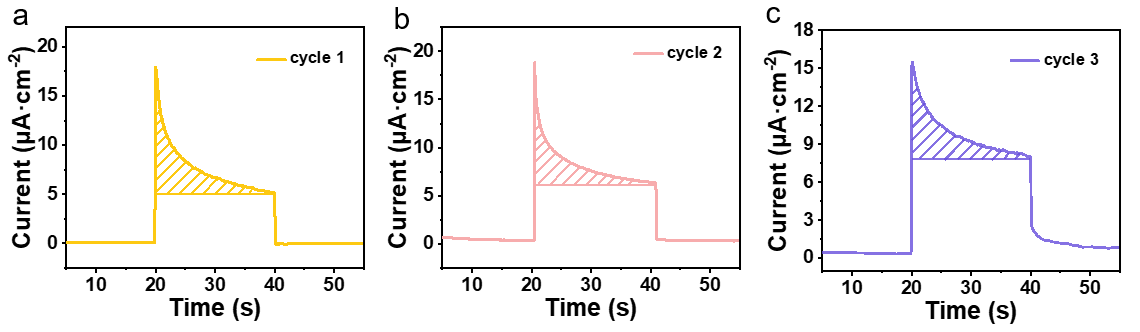


**Figure S39.** The photocurrent density for different number of cycles of LSA.


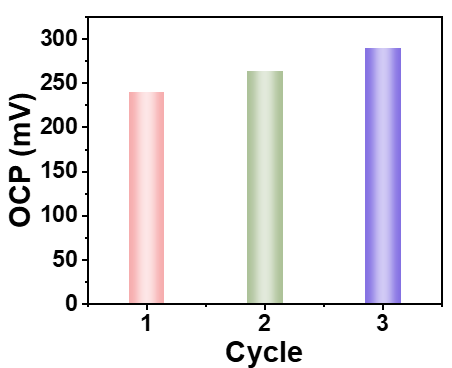


**Figure S40.** The open circuit potential for different number of cycles of LSA.


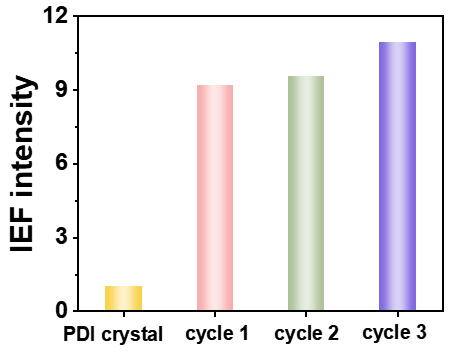


**Figure S41.** The IEF intensity for different number of cycles of LSA.


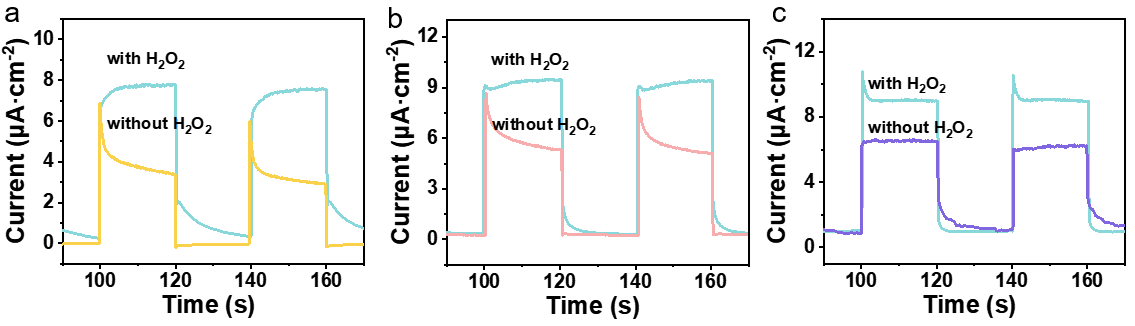


**Figure S42.** The transient photocurrent density with and without adding H_2_O_2_ of different number of cycles of LSA: (a) cycle 1, (b) cycle 2 and (c) cycle 3.


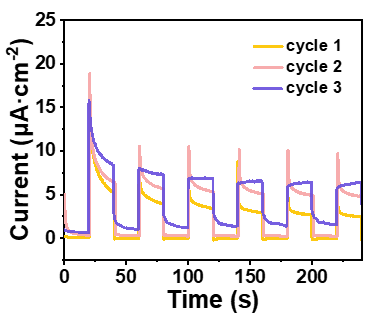


**Figure S43.** The photocurrent responses for different number of cycles of LSA.


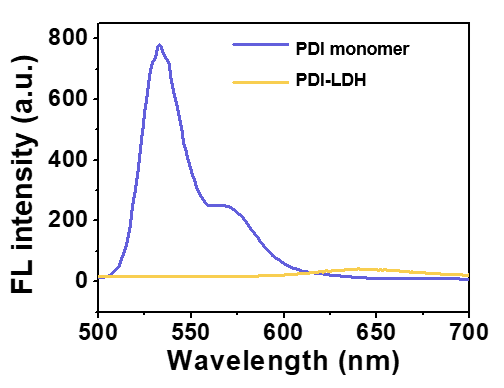


**Figure S44.** FL emission spectra of PDI monomer and PDI-LDH.


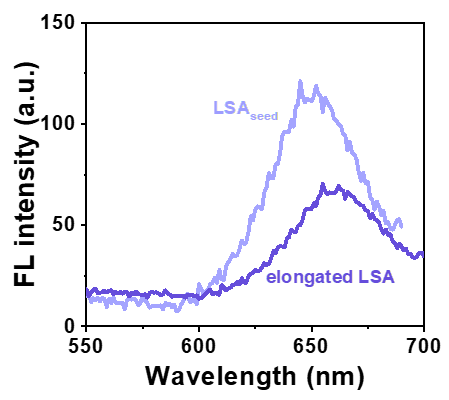


**Figure S45.** FL emission spectra of LSA_seed_ and elongated LSA.


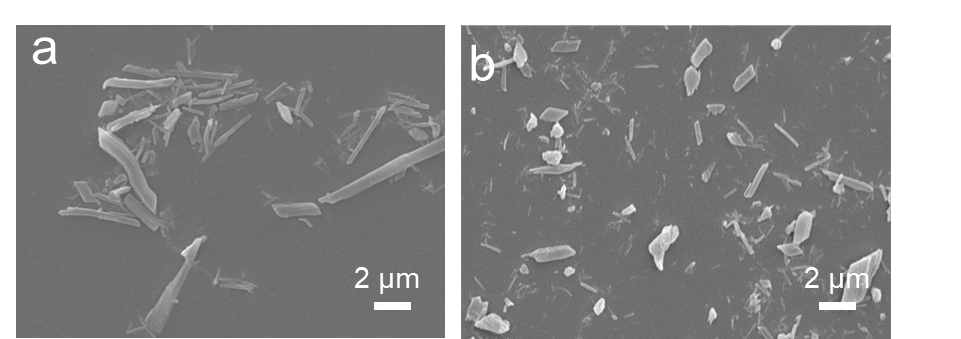


**Figure S46.** SEM images of (a) pure PDI (b) PDI + LDH dissolved in propionic acid/ CH_3_OH (1:3 v/v).


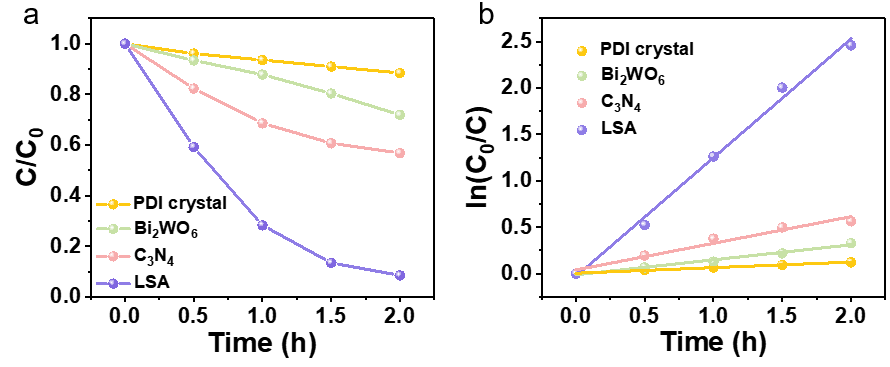


**Figure S47.** (a) Phenol photodegradation on PDI crystal, LSA, and other well-known photocatalysts, Bi_2_WO_6_ and C_3_N_4_. (b) The degradation kinetic curves of phenol by PDI crystal, Bi_2_WO_6_, C_3_N_4_ and LSA photocatalysts.


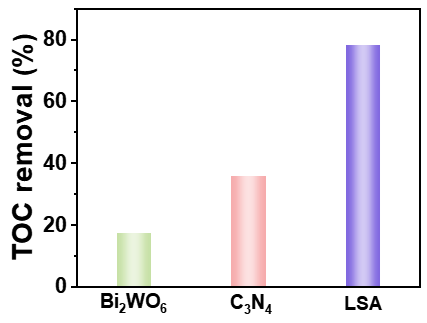


**Figure S48.** The TOC removal rate of phenol on Bi_2_WO_6_, C_3_N_4_ and LSA.


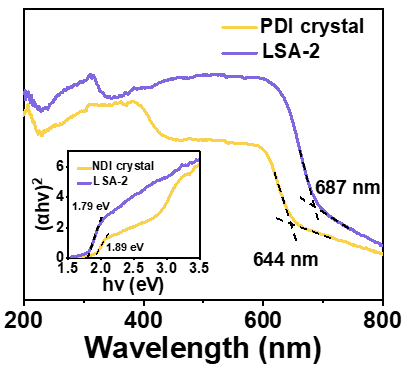


**Figure S49.** UV-vis diffuse reflectance spectrum of PDI crystal and LSA-2 (inset: band gap).


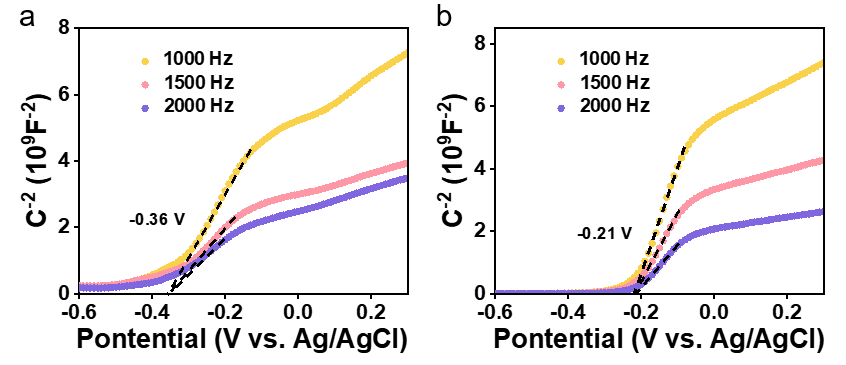


**Figure S50.** Mott-Schottky curves of (a) PDI crystal and (b) LSA-2.

The conduction band potential (*E*_CB_) is calculated from the equation *E*_CB_ (V vs. NHE) = *E*_fb_ (V vs. Ag/AgCl) + 0.210 – X, where X is the potential difference between the conduction band potential and the flat-band potential, which is generally positive by 0.1 eV compared to the flat-band potential for n-type semiconductors. The valence band potential (*E*_VB_) is calculated from the equation *E*_VB_ = *E*_CB_ + *E*_g_.


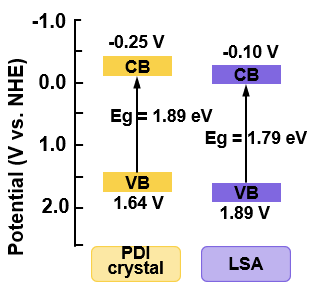


**Figure S51.** Energy Band Structure of the PDI crystal and LSA-2.


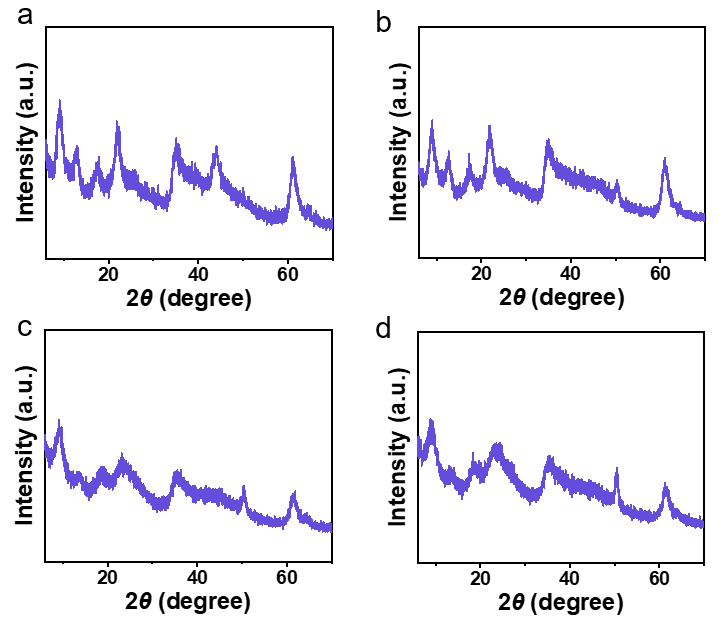


**Figure S52.** The XRD patterns of (a) NDI-B-LDH, (b) NDI-I-LDH, (c) PDI-B-LDH and (d) PDI-I-LDH.


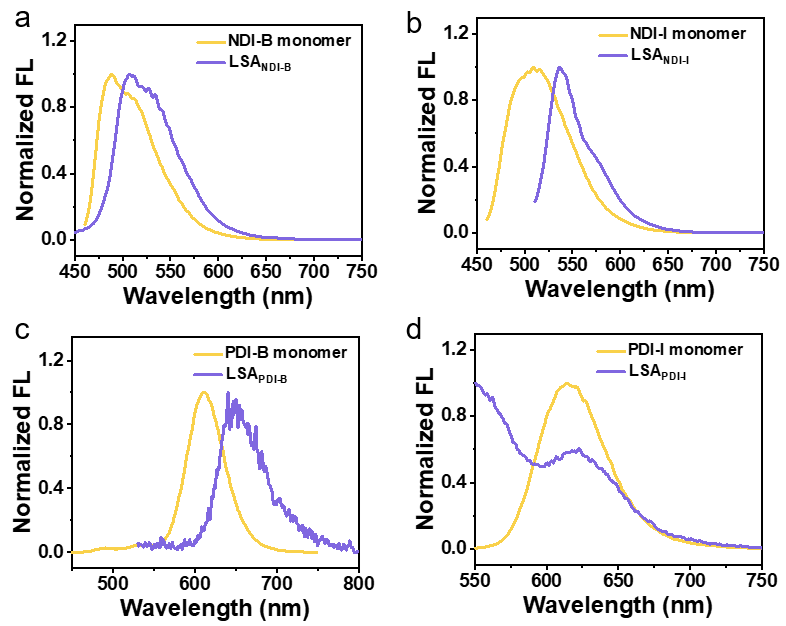


**Figure S53.** The FL spectra of (a) NDI-B monomer and LSA_NDI-B_, (b) NDI-I monomer and LSA_NDI-I_, (c) PDI-B monomer and LSA_PDI-B_ and (d) PDI-I monomer and LSA_PDI-I_.


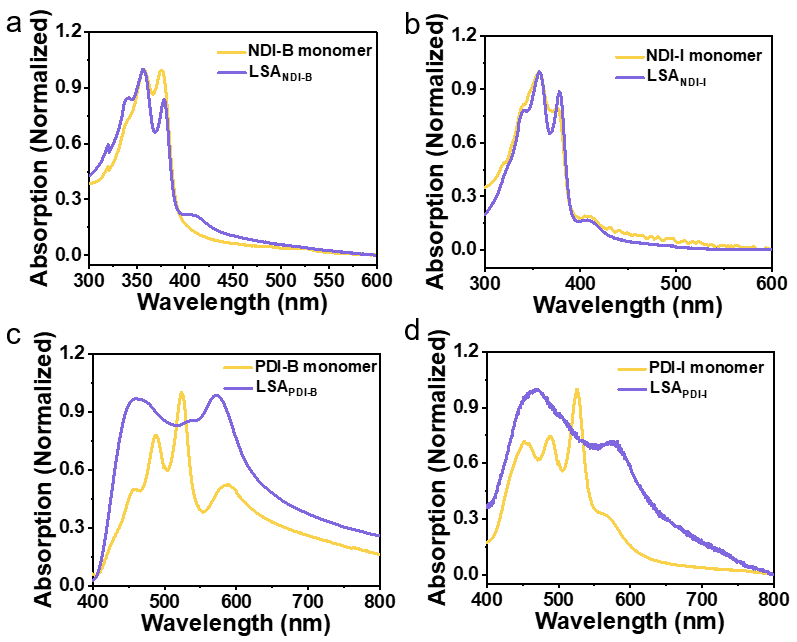


**Figure S54.** The UV-vis spectra of (a) NDI-B monomer and LSA_NDI-B_, (b) NDI-I monomer and LSA_NDI-I_, (c) PDI-B monomer and LSA_PDI-B_ and (d) PDI-I monomer and LSA_PDI-I_.


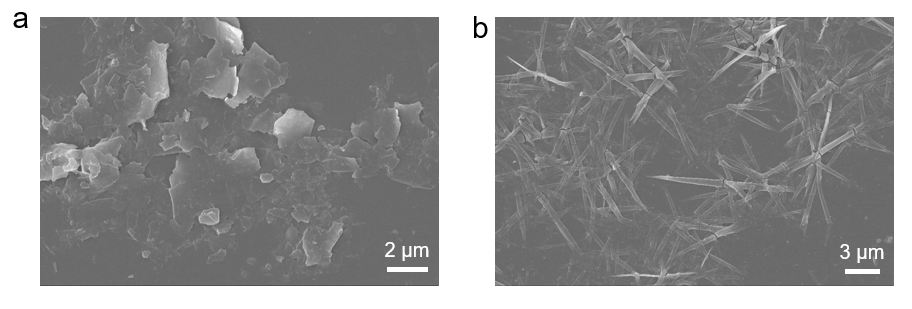


**Figure S55.** The SEM images of (a) NDI-B crystal and (b) LSA_NDI-B_.


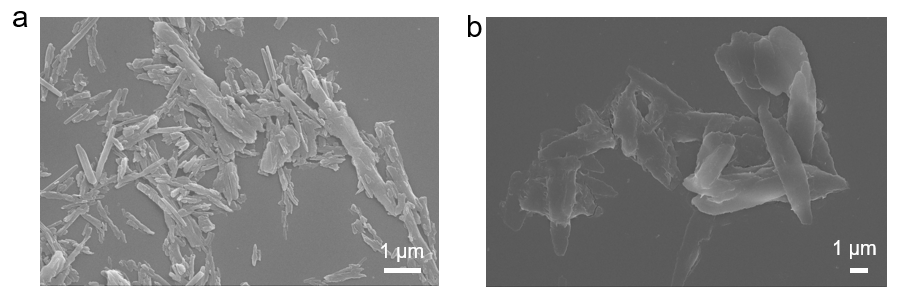


**Figure S56.** The SEM images of (a) NDI-I crystal and (b) LSA_NDI-I_.


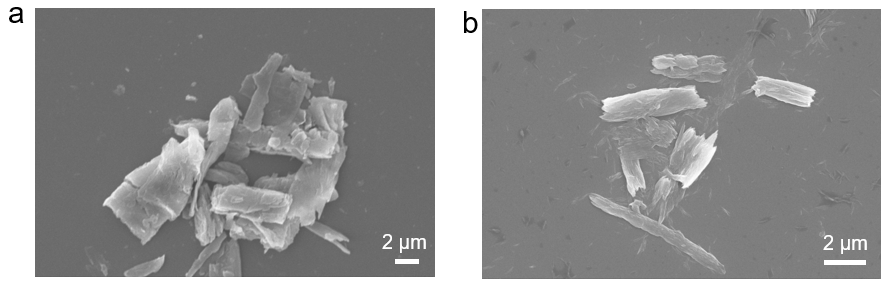


**Figure S57.** The SEM images of (a) PDI-B crystal and (b) LSA_PDI-B_.


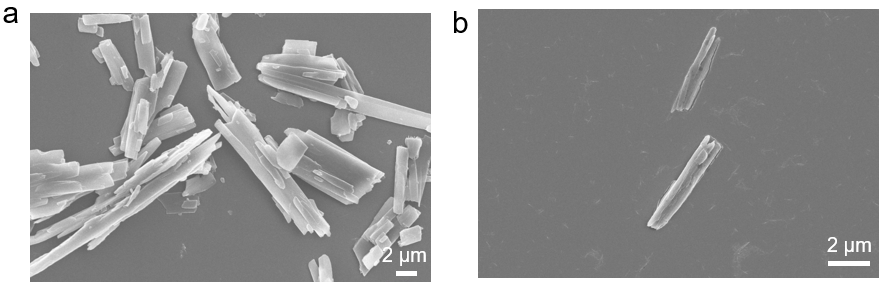


**Figure S58.** The SEM images of (a) PDI-I crystal and (b) LSA_PDI-I_.


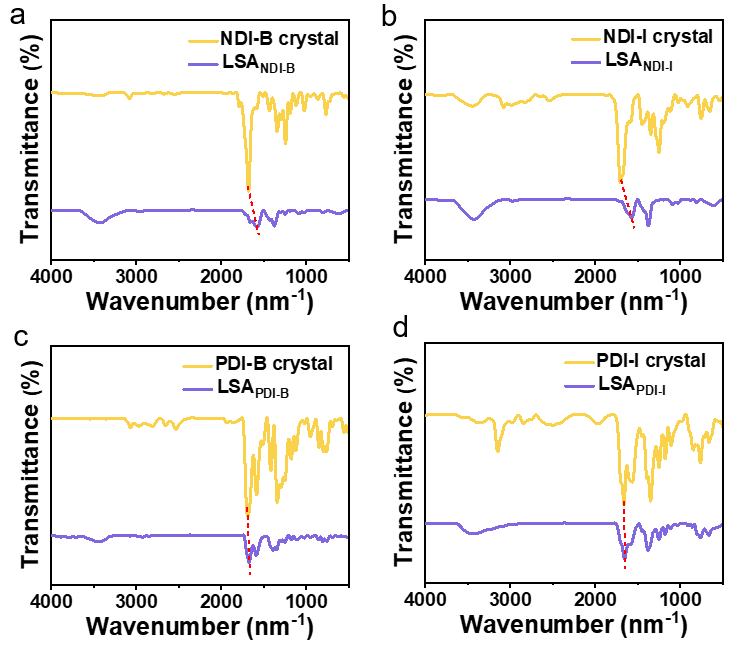


**Figure S59.** The FT-IR spectra of (a) NDI-B monomer and LSA_NDI-B_, (b) NDI-I monomer and LSA_NDI-I_, (c) PDI-B monomer and LSA_PDI-B_ and (d) PDI-I monomer and LSA_PDI-I_.

**
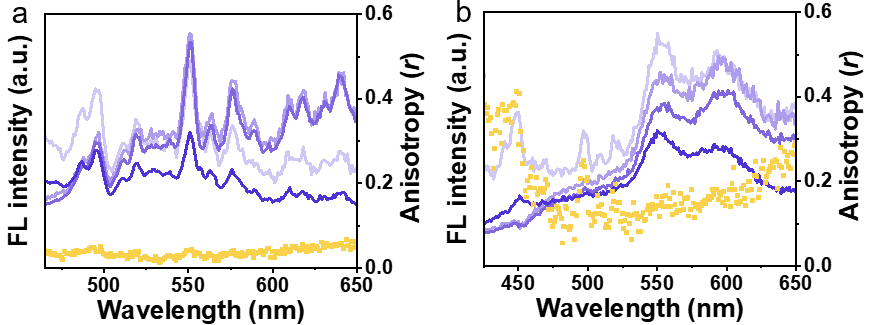
**

**Figure S60.** The polarization spectra and anisotropic profiles for (a) NDI-B crystal (*r* = 0.03) and (b) LSA_NDI-B_ (*r* = 0.17).

**
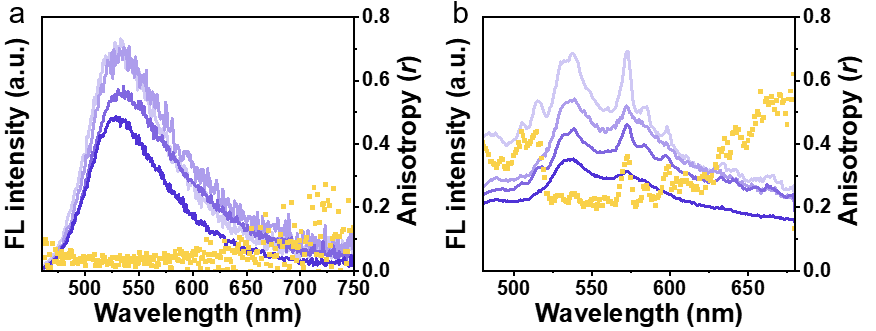
**

**Figure S61.** The polarization spectra and anisotropic profiles for (a) NDI-I crystal (*r* = 0.03) and (b) LSA_NDI-I_ (*r* = 0.17).

**
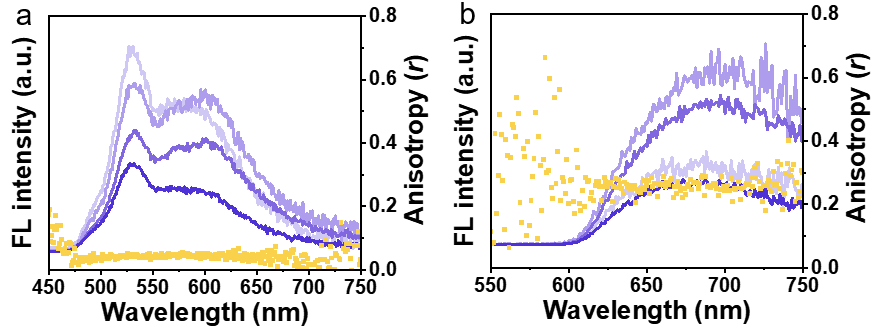
**

**Figure S62.** The polarization spectra and anisotropic profiles for (a) PDI-B crystal (*r* = 0.04) and (b) LSA_PDI-B_ (*r* = 0.26).

**
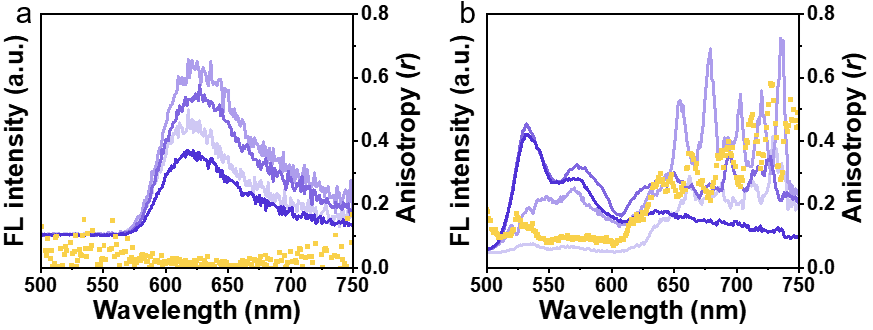
**

**Figure S63.** The polarization spectra and anisotropic profiles for (a) PDI-I crystal (*r* = 0.02) and (b) LSA_PDI-I_ (*r* = 0.25).


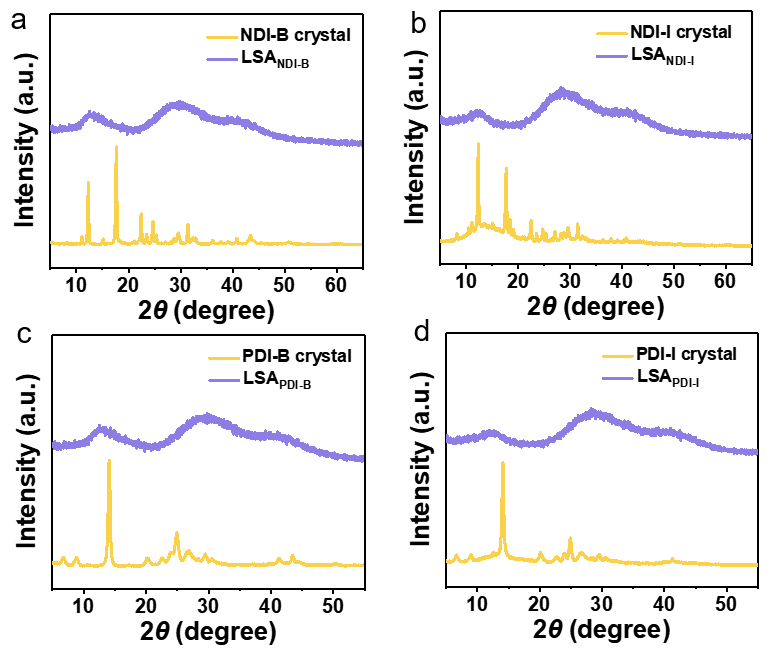


**Figure S64.** XRD patterns of (a) NDI-B crystal and LSA_NDI-B_, (b) NDI-I crystal and LSA_NDI-I_, (c) PDI-B crystal and LSA_PDI-B_, (d) PDI-I crystal and LSA_PDI-I_.


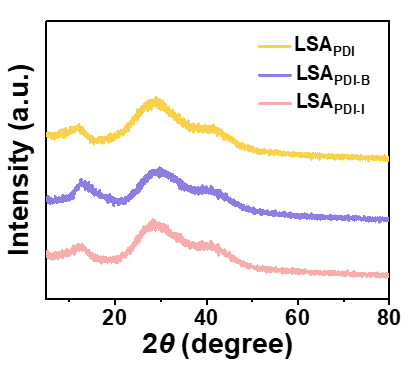


**Figure S65.** The XRD patterns of LSA_PDI_, LSA_PDI-B_ and LSA_PDI-I_.


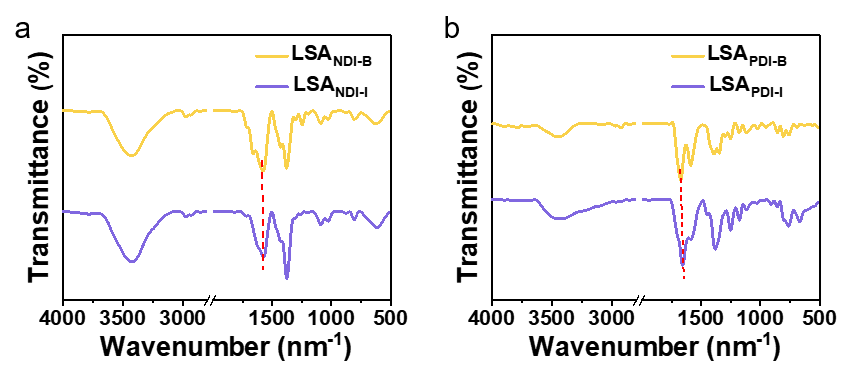


**Figure S66.** (a) The FT-IR spectra of LSA_NDI-B_ and LSA_NDI-I_. (b) The FT-IR spectra of LSA_PDI-B_ and LSA_PDI-I_.


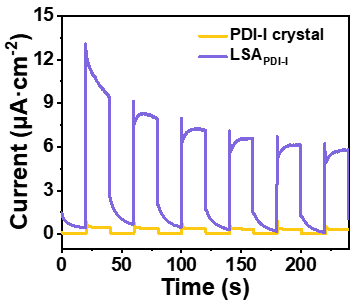


**Figure S67.** The photocurrent density of PDI-I crystal and LSA_PDI-I_.


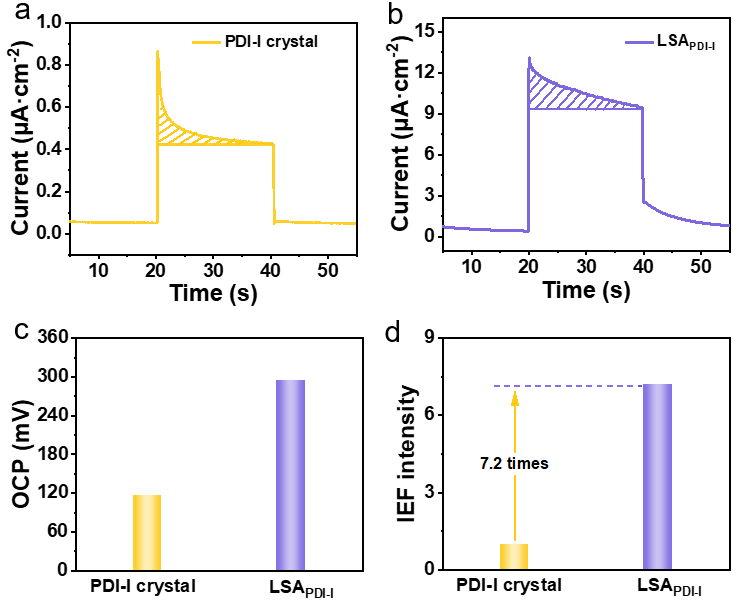


**Figure S68.** The photocurrent density of (a) PDI-I crystal and (b) LSA_PDI-I_. (c) The open circuit potential of PDI-I crystal and LSA_PDI-I_. (d) The relative IEF intensity of PDI-I crystal and LSA_PDI-I_.


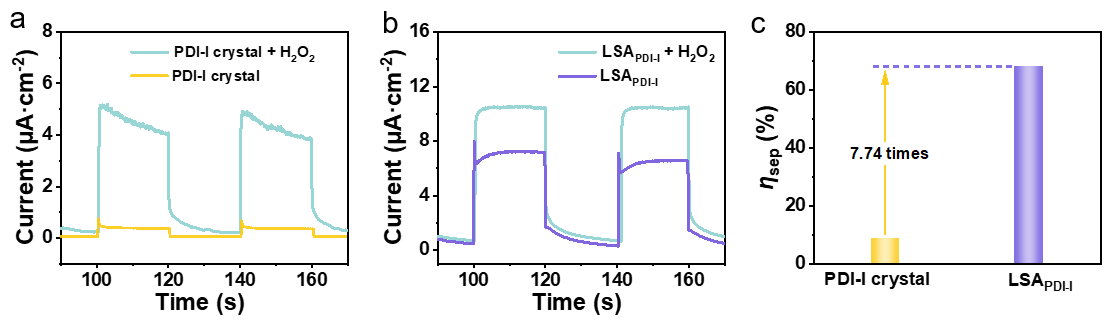


**Figure S69.** The transient photocurrent density with and without adding H_2_O_2_ of (a) PDI-I crystal and (b) LSA_PDI-I_. (c) The comparison of charge separation efficiency of PDI-I crystal and LSA_PDI-I_.


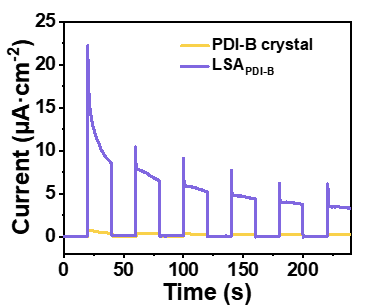


**Figure S70.** The photocurrent density of PDI-B crystal and LSA_PDI-B_.


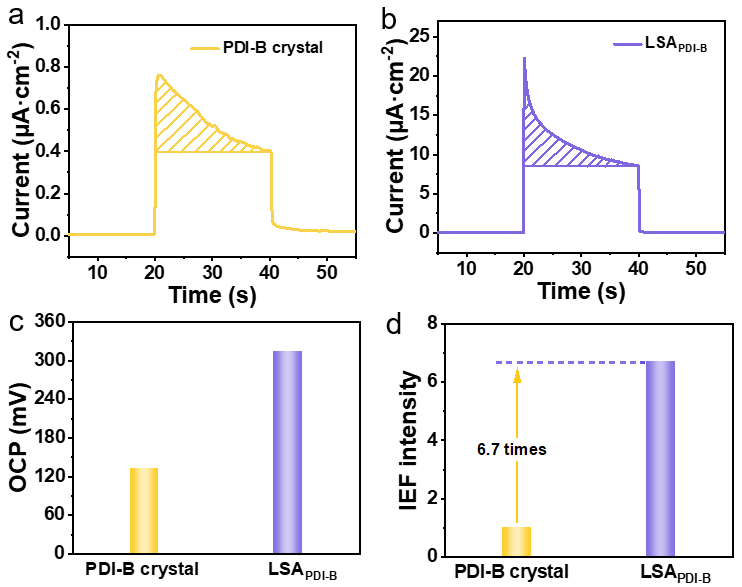


**Figure S71.** The photocurrent density of (a) PDI-B crystal and (b) LSA_PDI-B_. (c) The open circuit potential of PDI-B crystal and LSA_PDI-B_. (d) The relative IEF intensity of PDI-B crystal and LSA_PDI-B_.


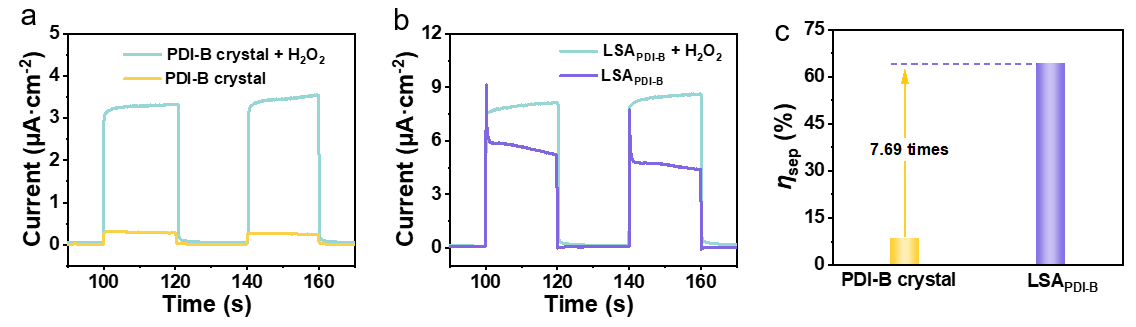


**Figure S72.** The transient photocurrent density with and without adding H_2_O_2_ of (a) PDI-B crystal and (b) LSA_PDI-B_. (c) The comparison of charge separation efficiency of PDI-B crystal and LSA_PDI-B_.


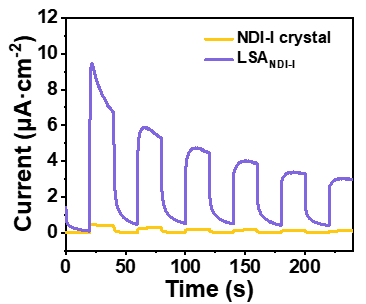


**Figure S73.** The photocurrent density of NDI-I crystal and LSA_NDI-I_.


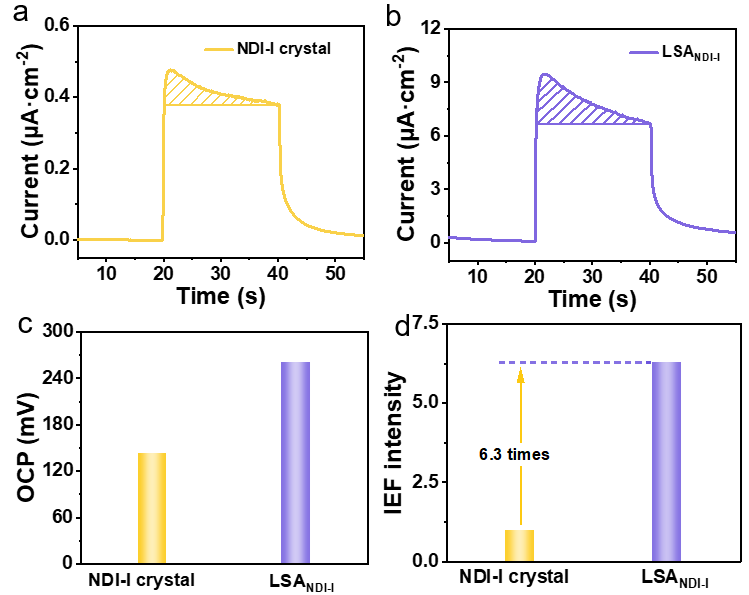


**Figure S74.** The photocurrent density of (a) NDI-I crystal and (b) LSA_NDI-I_. (c) The open circuit potential of NDI-I crystal and LSA_NDI-I_. (d) The relative IEF intensity of NDI-I crystal and LSA_NDI-I_.


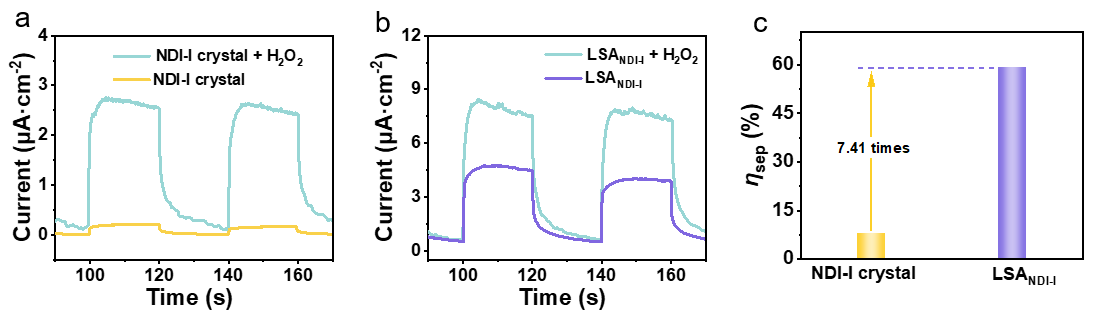


**Figure S75.** The transient photocurrent density with and without adding H_2_O_2_ of (a) NDI-I crystal and (b) LSA_NDI-I_. (c) The comparison of charge separation efficiency of NDI-I crystal and LSA_NDI-I_.


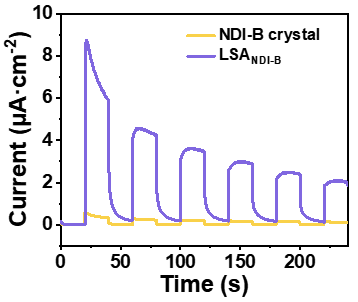


**Figure S76.** The photocurrent density of NDI-B crystal and LSA_NDI-B_.


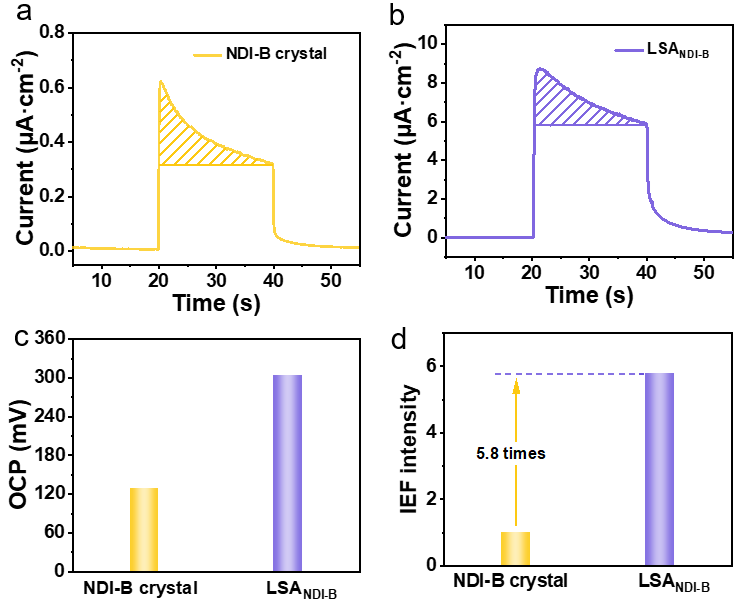


**Figure S77.** The photocurrent density of (a) NDI-B crystal and (b) LSA_NDI-B_. (c) The open circuit potential of NDI-B crystal and LSA_NDI-B_. (d) The relative IEF intensity of NDI-B crystal and LSA_NDI-B_.


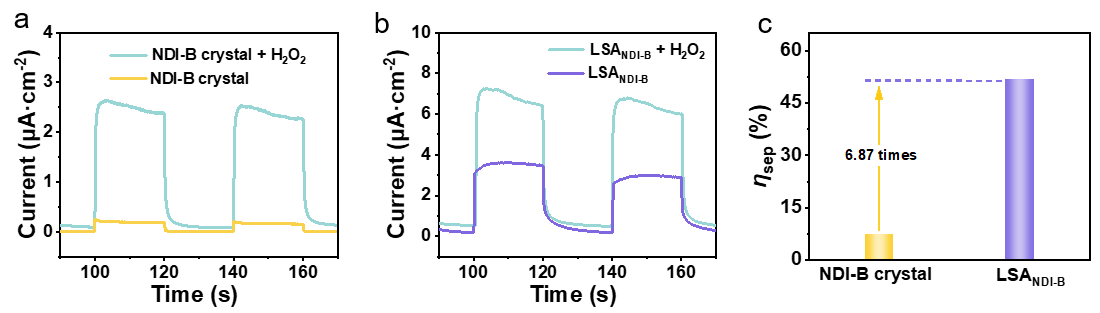


**Figure S78.** The transient photocurrent density with and without adding H_2_O_2_ of (a) NDI-B crystal and (b) LSA_NDI-B_. (c) The comparison of charge separation efficiency of NDI-B crystal and LSA_NDI-B_.


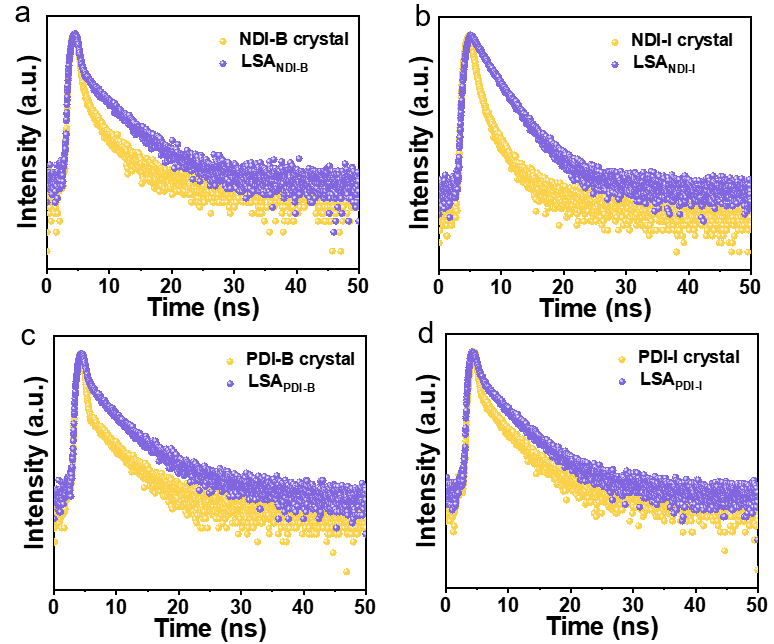


**Figure S79.** The time-resolved fluorescence spectra of (a) NDI-B crystal and LSA_NDI-B_, (b) NDI-I crystal and LSA_NDI-I_, (c) PDI-B crystal and LSA_PDI-B_, (d) PDI-I crystal and LSA_PDI-I_.


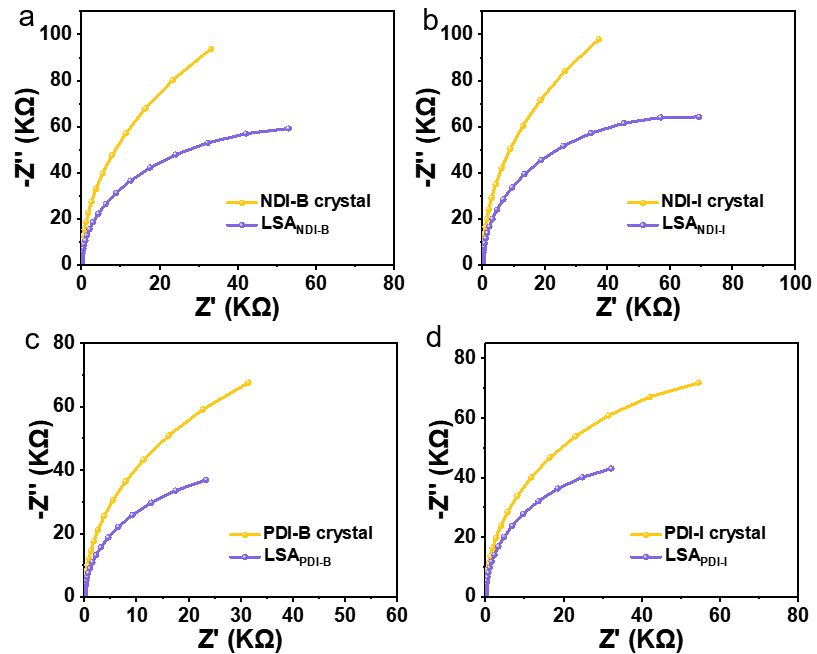


**Figure S80.** The electrochemical impedance spectra of (a) NDI-B crystal and LSA_NDI-B_, (b) NDI-I crystal and LSA_NDI-I_, (c) PDI-B crystal and LSA_PDI-B_, (d) PDI-I crystal and LSA_PDI-I_.

**Table S1.** The ICP-MS results of LDH, the calculated ratio of Mg/Al and corresponding chemical formula.

| Various LDHs | Mg (ppm) | Al (ppm) | Mg/Al | [Mg*_a_*Al*_b_*(OH)*_x_*](A*^n^*^−^)*_y_*∙*z*H_2_O | |
| --- | --- | --- | --- | --- | --- |
|  |  |  |  | *a* | *b* |
| PDI-LDH | 3.98 | 1.41 | 3.12 | 0.76 | 0.24 |

**Table S2.** The elemental analysis of intercalated LDHs, the calculated weight fraction of intercalated molecules and corresponding chemical formula.

| Various LDHs | N  (wt.%) | C  (wt.%) | H  (wt.%) | O  (wt.%) | A*^n^*^−^  (wt.%) | [Mg*_a_*Al*_b_*(OH)*_x_*](A*^n^*^−^)*_y_*∙*z*H_2_O | | |
| --- | --- | --- | --- | --- | --- | --- | --- | --- |
|  |  |  |  |  |  | *x* | *y* | *z* |
| PDI-LDH | 3.21 | 41.22 | 4.08 | 30.78 | 60.98 | 1.89 | 0.13 | 1.70 |

**Table S3.** Time-resolved fluorescence fitting results of PDI crystal LSA-1, LSA-2 and LSA-3.

| Samples | *τ*_1_ (ns) | B_1_ (%) | *τ*_2_ (ns) | B_2_ (%) | *τ*_avg_ (ns) |
| --- | --- | --- | --- | --- | --- |
| PDI crystal | 0.03296 | 75.82 | 1.401 | 24.18 | 0.59 |
| LSA-1 | 0.01777 | 58.69 | 1.848 | 41.31 | 0.87 |
| LSA-2 | 0.6153 | 27.62 | 4.185 | 72.38 | 3.20 |
| LSA-3 | 0.7047 | 40.50 | 2.710 | 59.50 | 1.90 |

**Table S4.** The π-π diffraction peak positions and intermolecular distances of universal LSAs and crystals.

| Samples | π-π stacking diffraction peak (°) | intermolecular distance (Å) |
| --- | --- | --- |
| NDI-B crystal | 24.70 | 3.60 |
| LSA_NDI-B_ | 28.67 | 3.11 |
| NDI-I crystal | 24.77 | 3.59 |
| LSA_NDI-I_ | 28.51 | 3.13 |
| PDI-B crystal | 24.89 | 3.57 |
| LSA_PDI-B_ | 28.24 | 3.15 |
| PDI-I crystal | 24.91 | 3.57 |
| LSA_PDI-I_ | 28.18 | 3.16 |

**Table S5.** Time-resolved fluorescence fitting results universal LSAs and crystals.

| Samples | *τ*_1_ (ns) | B_1_ (%) | *τ*_2_ (ns) | B_2_ (%) | *τ*_avg_ (ns) |
| --- | --- | --- | --- | --- | --- |
| NDI-B crystal | 0.5732 | 67.05 | 3.682 | 32.95 | 1.60 |
| LSA_NDI-B_ | 0.5477 | 30.43 | 4.401 | 69.57 | 3.23 |
| NDI-I crystal | 0.7823 | 65.42 | 3.197 | 34.58 | 1.62 |
| LSA_NDI-I_ | 2.526 | 53.91 | 4.207 | 46.09 | 3.30 |
| PDI-B crystal | 0.5343 | 60.61 | 4.598 | 39.39 | 2.14 |
| LSA_PDI-B_ | 0.7301 | 28.84 | 4.577 | 71.16 | 3.47 |
| PDI-I crystal | 0.4716 | 36.83 | 3.941 | 63.17 | 2.66 |
| LSA_PDI-I_ | 0.7593 | 23.36 | 4.400 | 76.64 | 3.55 |
